# Peritoneal metastasis of colorectal cancer (pmCRC): identification of predictive molecular signatures by a novel preclinical platform of matching pmCRC PDX/PD3D models

**DOI:** 10.1186/s12943-021-01430-7

**Published:** 2021-10-21

**Authors:** Mathias Dahlmann, Guido Gambara, Bernadette Brzezicha, Oliver Popp, Eva Pachmayr, Lena Wedeken, Alina Pflaume, Margarita Mokritzkij, Safak Gül-Klein, Andreas Brandl, Caroline Schweiger-Eisbacher, Philipp Mertins, Jens Hoffmann, Ulrich Keilholz, Wolfgang Walther, Christian Regenbrecht, Beate Rau, Ulrike Stein

**Affiliations:** 1 Translational Oncology of Solid Tumors, Experimental and Clinical Research Center, Charité - University Medicine Berlin, and Max-Delbrück-Center for Molecular Medicine in the Helmholtz Association, Robert-Rössle-Str. 10, 13125 Berlin, Germany; 2 German Cancer Consortium (DKTK), Heidelberg, im Neuenheimer Feld 280, 69120 Heidelberg, Germany; 3 Charité Comprehensive Cancer Center, Charité – Universitätsmedizin Berlin, Invalidenstr. 80, 10117 Berlin, Germany; 4 EPO GmbH Berlin-Buch, Robert-Rössle-Str. 10, 13125 Berlin, Germany; 5 Proteomics Platform, Max-Delbrück-Center for Molecular Medicine and Berlin Institute of Health, Robert-Rössle-Str. 10, 13125 Berlin, Germany; 6 Department of Surgery, Charité - University Medicine Berlin, Augustenburger Platz 1, 13353 Berlin, Germany; 7 CELLphenomics GmbH, Robert-Rössle-Str. 10, 13125 Berlin, Germany; 8 Institute of Pathology, University Medicine Göttingen, Robert-Koch-Str. 40, 37075 Göttingen, Germany

## Main text

Colorectal cancer (CRC) is the third most frequent cancer type worldwide [1] and distant metastasis represents its most lethal attribute. About every second CRC patient develops distant metastasis [2, 3] and about 30% as peritoneal metastasis (pmCRC) [4] associated with inferior outcome and limited treatment opportunities [5, 6]. This defines an urgent need for applied translational research to identify and exploit new biomarkers, signatures, and molecular targets for personalized pmCRC treatment with well-characterized pre-clinical disease models.

**Fig. 1:**
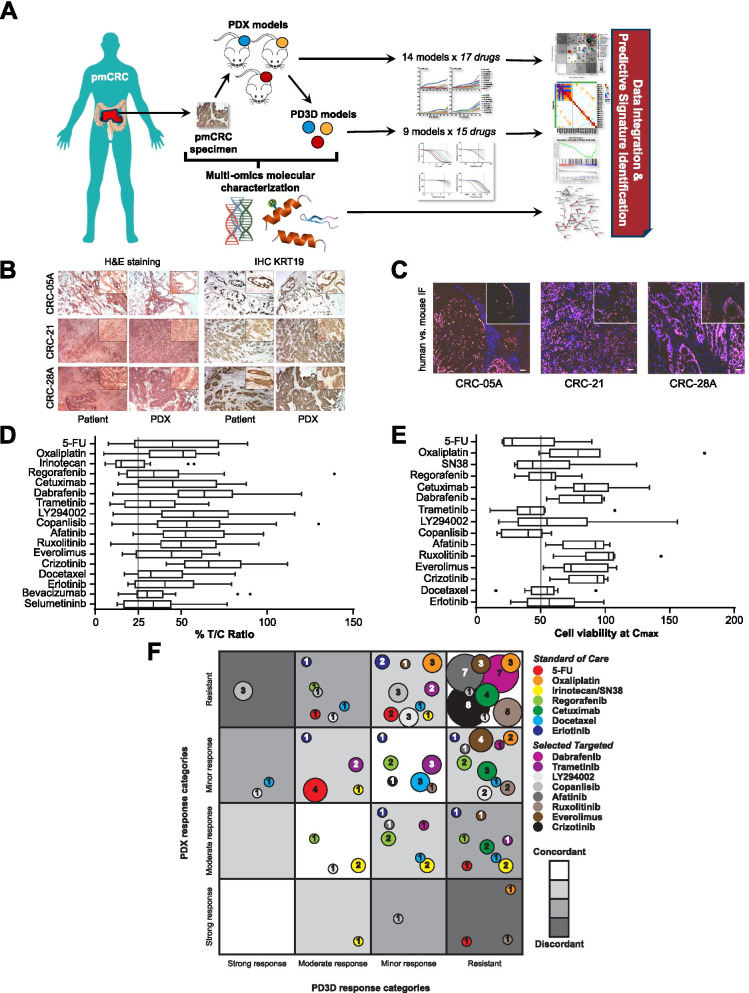
Matched pmCRC models retain histopathological tumor features and are suitable to determine therapeutic response. **A** Schematic representation of the project to generate a preclinical platform of matched pmCRC PDX/PD3D models for evaluating treatment response and predictive biomarker signatures. **B** PDX tumors retain histopathological features of the original human metastastic tissue, determined by H&E staining and KRT19 immunohistochemistry. **C** Human tumor stroma in pmCRC PDX models is replaced by mouse stroma during passaging, determined by immunofluorescence. **D** Treatment response of pmCRC PDX models (*n* = 14). Treatment of PDX models was started at palpable tumors (0.1 cm^3^) and the ratio of the mean TV of the treated group (T) and the solvent treated control group (C) was expressed as the T/C-value in percent. **E** Treatment response of pmCRC PD3D cell cultures (*n* = 9) as viability at highest plasma concentration (C_max_) of each tested compound. Whiskers and outliers are plotted according to Tukey. **F** Bubble plot representation of categorized treatment response of 9 matched pmCRC PDX and PD3D models (T/C and viability at C_max_, respectively) for treatment with SoC and targeted drugs. The shading of the fields indicates the degree of concordance in response, the color of each circle represents individual drugs and the size of each circle corresponds with the number of models in the same category. T/C-values for PDX models were categorized as strong response (0–10%), moderate response (11–25%), minor response (26–50%) and resistant (> 50%). Similarly, viability of PD3D cell cultures at C_max_ was categorized as strong response (0–30%), moderate response (31–60%), minor response (61–80%) and resistant (> 80%)

Here we report newly established matched PDX and PD3D pmCRC models as molecularly characterized platform for pre-clinical and co-clinical evaluation of treatment response and identification of predictive biomarkers (Fig. 1A). We received 57 surgical specimens from 37 pmCRC patients and established 14 pmCRC PDX models from 10 patients (see Table S1). Nine PDX models were derived from pmCRC at the peritoneum and five from the omentum, with four model pairs from both sites of the same patient. The mean tumor doubling time of the PDX models was 10.9 ± 6.2 d, ranging from 4.2 d to 28.4 d, with significantly different growth rates for two PDX pairs (Fig. S1A). Histological comparison of patient metastases with corresponding PDX tumors revealed similar features of adenocarcinoma (Fig. 1B). Further, PDX tumors were positive for human nuclei antibody staining, leaving surrounding stroma negative. This indicates replacement of human by murine stroma during in vivo passaging (Fig. 1C). The majority of PDX tumors contained about 5% to 15% murine stroma, while two models showed up to 40% mouse stroma (Table S6). To generate matched PD3D models, 13 PDX tumors have been explanted and processed, as described by Schütte et al. [7], succeeding in establishing nine pmCRC PD3D models.

PDX and PD3D models were treated with standard-of-care (SoC) and targeted drugs with individual concentrations and application schemes (Table S2). Within PDX models, irinotecan showed best response for SoC drugs, while MEK inhibition (trametinib, selumetinib) showed best response for targeted treatment (Fig. 1D; Fig. S1B,C; Table S3). Interestingly, only one model showed treatment response to both trametinib and selumetinib, even within models from the same patient, possibly reflecting their individual modes of action in MEK1 inhibition [8]. Similarly, 5-FU and SN38 treatment, respectively, resulted in robust growth inhibition of PD3D models, while best efficacy among targeted drugs was observed for PI3K and MEK inhibition (Fig. 1E; Tables S4,S5). By plotting the categorized responses for each drug, we observed 76 ± 20% of all matched PDX/PD3D models distributed in a range of moderate to high concordance (Fig. 1F). Highest number of concordant response of matched PDX/PD3D models to SoC treatment was observed for oxaliplatin (*n* = 8), followed by cetuximab, regorafenib and erlotinib (*n* = 7, each). Least response concordance was observed with irinotecan/SN38 (*n* = 5) and although the response of the PD3D cell culture models correlates with the expression pattern of SLCO1B3 as a SN83 transporter [9] and UGT1A1, which catalyzes the glucuronylation of SN38 [10], a molecular mechanism of the observed response discordance needs to be validated. In opposite, some targeted drugs showed poor response rates in both PDX and PD3D models, but with high concordance, which was verified by low respective pathway activity (Fig. S3A). Least concordance of PDX and PD3D model response to targeted drugs was observed for copanlisib (*n* = 2), which indicates altered PI3K signaling activity, bypassing or crosstalk of other signaling pathways within the respective model type. Taken together, although we observed rather discordant responses in the pmCRC models in some cases of treatment, the generation of matched preclinical models in general can identify best model types for response evaluation of individual therapies.

**Fig. 2:**
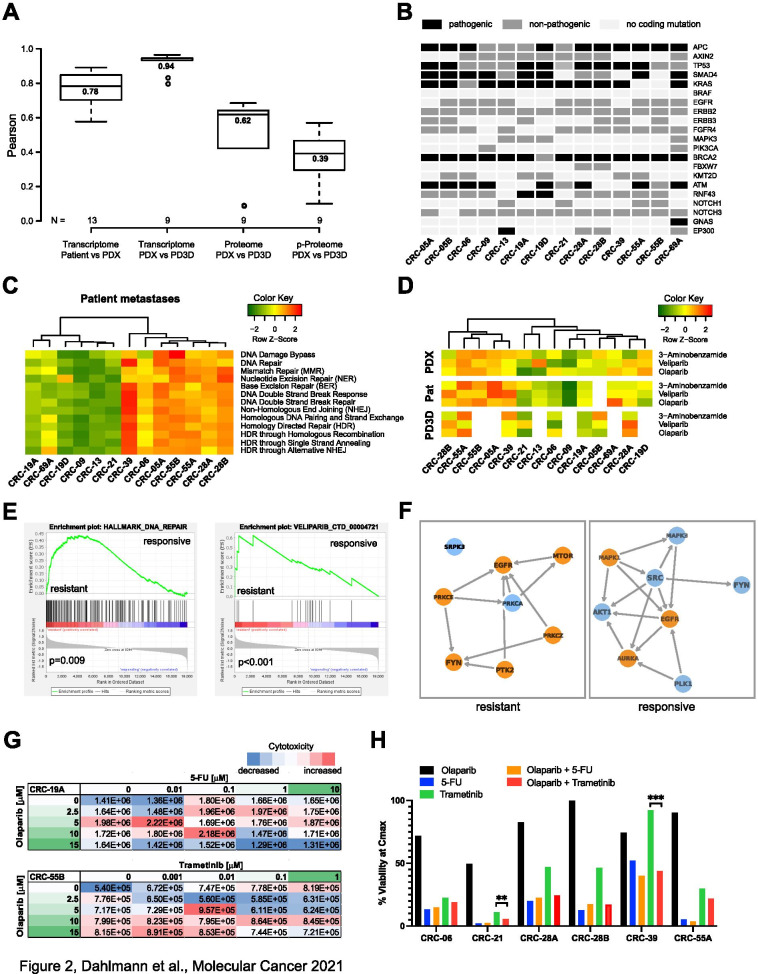
Multi-omics characterization of pmCRC metastases, PDX tumors and PD3D models confirms similarity of matched pmCRC models and can be used for predictive biomarker analysis. **A** Box plot representation of Pearson correlation coefficients of matched models on the basis of transcriptome, proteome and phosphoproteome analysis of patient metastases, PDX tumors and PD3D cell culture models. Center lines show the medians; box limits and whiskers are plotted according to Tukey, outliers are represented by dots. **B** Representation of the occurrence of frameshift mutations, truncations or amino acid substitutions commonly found in CRC. Respective gene mutations were counted when they were found in the patient metastasis or enriched in the generated PDX or PD3D models. Black – pathogenic according to ClinVar, dark grey – non-pathogenic/uncertain according to ClinVar, light grey – no alteration to reference sequence. **C** Single sample gene set enrichment analysis (ssGSEA) of expressed transcripts of patient metastases focusing on DNA repair signatures (MsigDB/Reactome). **D** Prediction of treatment response to PARP inhibitors 3-aminobenzamide, olaparib and veliparib by ssGSEA of drug response signatures (DsigDB) of expressed transcripts. **E** GSEA of PDX tumor expression signatures grouped for response or resistance to 5-FU treatment. Significant enrichments were found for DNA repair in general (MsigDB/Hallmarks) and the signature for response to veliparib treatment (DsigDB). **F** Visualization of altered kinase activity and interaction (KEA3) by integrated proteome and phosphoproteome data of 5-FU responsive and resistant PDX models. Blue – present in top-10 of either MeanRank or TopRank score, orange – present in both top-25 of both MeanRank and TopRank score. **G,H** Response evaluation of pre-clinical pmCRC models under combinatorial treatment of 5-FU or trametinib with olaparib. Explanted PDX tumor cells (**G**) were treated with the indicated drug concentration and combination for 24 h in the presence of a fluorescent cytotoxicity marker. Fluorescence signals of each treatment condition, indicating dead or dying cells, were normalized to the cell confluence of the same well (*n* = 2). Blue – decreased cytotoxicity compared to median, red – increased cytotoxicity compared to median. PD3D cell culture models (**H**) were treated with C_max_ concentrations of 5-FU, trametinib, olaparib or drug combinations and treatment response was determined as cell viability after 4 days (*n* = 4)

In general, by generating preclinical models, mainly human tumor cells are maintained in the PDX tumors and PD3D cell culture, which certainly undergo adaptation to their respective environment (in vitro culture or mouse), but maintain key molecular characteristics and sensitivity profiles. This is accompanied by the lack of transcripts specific for human tumor stroma in these samples. Although tumor stroma cells, and immune cells in particular, of patient tissues have an emerging prognostic and predictive value, they only marginally contribute to the treatment response in the established preclinical models. For identification of novel predictive biomarkers in pmCRC for SoC and targeted drug treatments we molecularly characterized the original patient pmCRC and corresponding PDX/PD3D models by RNAseq and patient-derived pmCRC models also by mass-spectrometry proteomics and phosphoproteomics. Transcript expression patterns and known polymorphisms correlated highly between matched patient metastases and PDX, but also between matched PDX/PD3D models, similarly to protein expression and phosphorylation (Fig. 2A; Fig. S2C-F). Classifying the biological features of pmCRC by predicting the consensus molecular subtype (CMS), which also impacts treatment decisions [11], resulted in subtype 4 for the majority of patient samples (Table S1). CMS 4 is characterized by a mesenchymal phenotype that reflects the predominant therapy resistance with partial response to irinotecan [12]. The analysis of genetic alterations commonly occurring in CRC confirmed the clinically determined KRAS-G12/13 mutation status of patients (Table S1), but also detected an additional pathogenic KRAS-Q61K mutation. Observed pathogenic mutations of APC, p53, SMAD4, RNF43, GNAS and EP300 are mainly maintained in the derived models (Fig. 2B, Table S7) and are similar to previously reported mutation rates for metastasized CRC (Table S9) [13, 14]. According to tumor heterogeneity, enrichment or loss of individual tumor cell types during model generation, some occurring cancer-related mutations were not detected in every sample of the respective model. For clinical application, relevant mutations in patient metastases should then be detected at higher precision, e.g. by targeted sequencing. Of note, we observed an unexpected high number of frameshift mutations in BRCA2 and further genes related to DNA damage repair, like ATM, ATR and CDK12 among the pmCRC samples (Fig. 2B; Tables S7, S8 and S9), compared to the much lower rate of BRCA1/2 mutations in MSS CRC (< 2%), which can rise to > 20% in MSI-H CRC [13]. As BRCA1/2 mutations are only marginally associated with successful PDX engraftment [15] and their mutation status is preserved from patient tissue over several PDX passages [16] we do not assume a biased model generation. Nevertheless, our findings strongly support further studies about the use of PARP inhibitors as treatment for pmCRC with the identified biomarker profile. In turn, analyzing the transcriptomes of patient metastases and derived models according to cancer hallmark gene signatures (including DNA repair in general), showed similar patterns of gene set enrichments at transcriptome and proteome level (Fig. S3A,B). When focusing on cellular DNA repair mechanisms in more detail, we observed clustering of patient metastases according to their predicted DNA repair activity (Fig. 2C). This was reflected in transcriptomic and proteomic analyses of PDX and PD3D models (Fig. S4A,B). This pattern was again observed when all sample types were predicted for their response to selected PARP inhibitors (Fig. 2D, Fig. S5A,B). The enrichment of DNA damage repair pathways in individual samples was analyzed in more detail by selecting pathway-specific gene sets for base and nucleotide excision repair, homologous recombination and Fanconi anemia [17] (Fig. S6A,B). Furthermore, gene set enrichment analysis (GSEA) of combined PDX models showing treatment response to 5-FU versus resistant models resulted in significantly enriched gene sets indicating DNA repair (ES = 0.44, *p* = 0.009), specifically NER (ES = 0.42, *p* = 0.002), and response to veliparib (ES = 0.62, *p* < 0.001; Fig. 2F, Fig. S7A). Similarly, PDX models resistant to selumetinib treatment showed enriched gene signatures for BER (ES = 0.57, *p* = 0.016), Fanconi anemia pathway (ES = 0.46, *p* = 0.061) and veliparib response (ES = 0.51, *p* = 0.004; Fig. S7B). Metascape and Kinase Enrichment Analysis [18, 19] were used to analyze integrated proteomic and phosphoproteomic data of grouped resistant and responsive models. Differential 5-FU response of PDX models was mainly characterized by altered α6/β4 signaling (Fig. S7C), with differential activity of PKC, PTK2/FAK and FYN (Fig. 2F, Fig. S4C, Fig. S7D,E). PTK2/FAK signaling has been recently connected to DNA damage response regulation [20]. Phospho-ɣ-H2AX, as an indicator of DNA double-strand breaks [21], has been found significantly less abundant in 5-FU resistant PDX models (log_2_FC = − 1.91, *p* = 0.002). As PARP activity is found in virtually all DNA repair mechanisms [22], its inhibition in tumor cells with a deficiency in homologous recombination (e.g. mutated BRCA1/2) leads to cell death and besides its clinical use in treating ovarian and breast cancer, it is also evaluated for gastrointestinal tumors [23–25]. Recent reports demonstrate the synergistic effect of combining PARP inhibitors with 5-FU in CRC treatment [26, 27]. Similarly, combined inhibition of PARP and MEK represents a promising rationale for novel anti-cancer therapy [28], which is already tested in a clinical phase I trial (NCT03162627). For response analysis of combination treatment of the PARP inhibitor olaparib with either 5-FU or trametinib in vitro, we selected pmCRC models according to the list of identified predictive biomarkers (Table S12) and employed different approaches: first we used single cell suspensions of PDX tumor tissues, applied a drug concentration matrix (Fig. 2G) and measured cell cytotoxicity over time. Indeed, we found a synergistic effect of both 5-FU and trametinib treatment in combination with olaparib in resistant models, compared to models that already responded well to the individual drug alone (Fig. 2G, Fig. S8A-D, Table S12). Second, treatment of PD3D models was performed similarly and confirmed the improved response to combination therapy of 5-FU or trametinib with olaparib (Fig. 2H, Fig. S8E, Table S12).

Analysis of further factors, such as age, sex, localization of the primary CRC (left/right colon), the localization of the peritoneal metastasis (peritoneum/omentum) or its histopathology (mucinous/non-mucinous adenocarcinoma) for treatment response to the tested SoC and targeted drugs, revealed no statistically significant predictive impact.

In summary, together with DNA repair deficiency promising novel predictive biomarkers were identified by molecular characterization of the pmCRC models, mainly analyzing differential gene expression of responders and non-responders for each drug treatment. Sensitivity and specificity of response prediction using ROC-based cut-off values for PDX and patient metastases resulted in matching biomarkers for the respective treatment response (Table S10), similarly to potentially predicting transcript variants (Table S11), ready to be included in prospective studies.

## Conclusion

This study reports for the first time the establishment of matched PDX/PD3D models from pmCRC, including thorough molecular characterization by multi-omics. Predictive biomarkers were identified for pmCRC to facilitate treatment selection for improved outcome. One of the novel key finding is the high occurrence of mutation in genes encoding for homologous recombination enzymes in almost all analyzed pmCRC patient samples, but activated alternative DNA repair mechanisms in samples resistant to 5-FU or MEK inhibitors. Pre-clinical pmCRC models resistant to the individual 5-FU or trametinib monotherapy showed an improved response in combination therapy with olaparib. This encourages the evaluation of PARP inhibitors, either as monotherapy in pmCRC or in combination with DNA damage-inducing drugs or MEK inhibition, for more effective pmCRC treatment. Thus, our pmCRC models are not only of value for advanced prognosis but also for tailoring therapies based on molecular characteristics of pmCRC as new momentum for clinical translation.

## Supplementary Information

**Additional file 1: Figure S1.** Growth and treatment response of pmCRC PDX models. A) Tumor doubling time of untreated pmCRC PDX models was assessed by volumetric measurement of the tumor growth in two dimensions with a caliper. Tumor volumes (TV) were determined by the formula: TV = (width^2^ x length) × 0.5, and show variances between models of the same patient, but different localization (*n* = 3). Bold – CRC metastasis localized at the peritoneum, regular – CRC metastasis localized at the omentum. B-C) Treatment response of pmCRC PDX models to individual SoC (B) and selected targeted drugs (C).

**Additional file 2: Figure S2.** Growth and treatment response of pmCRC PD3D models and high correlation of molecular characteristics indicate high similarity of each generated pair of matched pmCRC PDX and PD3D cell culture models. A) Representative images showing the effect of the tested compounds on the size and morphology of PDX-derived PD3Ds (exemplified by CRC-21). Scale bar = 100 μm, B) Dose-response fitted curves showing cell viability after 4 days (*n* = 4) of the different PD3D models for each compound tested. Dotted lines show the maximum human plasma concentration of each tested drug (C_max_). C-F) Distributions of Pearson and Spearman correlation values, comparing pmCRC metastases (red bars), PDX tumors (green bars) and PD3D cell culture models (blue bars) after transcriptomic (C,D), proteomic (E) and phosphoproteomic (F) analysis.

**Additional file 3: Figure S3.** Single sample gene set analysis (ssGSEA) confirms similarity of pmCRC patient metastases and derived matched models in predicted activity of cancer-related cellular processes. A) Transcriptomic single sample enrichment analysis of cancer hallmark gene sets of pmCRC metastases, PDX tumors and PD3D culture cell models. B) Proteomic single sample enrichment analysis for cancer hallmark signatures of pmCRC PDX tumor and PD3D cell culture models.

**Additional file 4: Figure S4.** Patient samples and derived pre-clinical models are similar in their predicted activity of cellular DNA repair mechanisms and signaling pathways. A) Transcriptomic single sample enrichment analysis of reactome gene sets related to DNA repair of pmCRC metastases, PDX tumors and PD3D culture cell models. B) Proteomic single sample enrichment analysis for DNA repair signatures of pmCRC PDX tumor and PD3D cell culture models. C) Single sample enrichment analysis of phosphoproteomic signaling pathway signatures. PSP – PhosphoSitePlus, P100 – PanoramaWeb/LINCS, NP – NetPath.

**Additional file 5: Figure S5.** Single sample gene set analysis confirms similarity of pmCRC patient metastases and derived matched models in predicted response to anti-cancer drugs. A) Transcriptomic single sample enrichment analysis of signatures predicting treatment response to selected anti-cancer drugs of pmCRC metastases, PDX tumors and PD3D culture cell models. B) Proteomic single sample enrichment analysis for drug response signatures of pmCRC PDX tumor and PD3D cell culture models.

**Additional file 6: Figure S6.** Pathway-specific gene sets reveal less active DNA damage repair pathways in pmCRC patient samples compared to preclinical models. A,B) Transcriptomic single sample enrichment analysis of pathway-specific gene sets predicting the activity of individual DNA damage repair pathways (A) and the expression distribution of pathway-specific gene expression (B). Pat – pmCRC patient metastases.

**Additional file 7: Figure S7.** Integrated analysis of altered signatures of cellular processes, signaling pathway and kinase activity in treatment resistant and responsive PDX models. A,B) GSEA of PDX tumor transcript expression signatures grouped for response or resistance to 5-FU (A) and selumetinib (B) treatment. Significant enrichments were found for DNA repair in general (MsigDB/Hallmarks), nucleotide and base excision repair, as well as Fanconi anemia (MsigDB/Reactome) and the signature for response to veliparib treatment (DsigDB). C) Visualization of altered cellular processes (Metascape) according to integrated proteome and phosphoproteome data of 5-FU responsive and resistant PDX models. D,E) Integrated proteome and phosphoproteome data analysis (KEA3) of 5-FU resistant (D) and responsive (E) PDX models for altered kinase activity and visualization of interaction networks. Left panels list the top-10 kinases according to their sum of ranks (MeanRank score), with colors indicating the scores used from external sources. Middle panels list the top-10 kinases according to their TopRank score. Right panels visualize the interaction networks of top scoring kinases for each analysis. Blue – present in top-10 of either MeanRank or TopRank score, orange – present in both top-25 of both MeanRank and TopRank score.

**Additional file 8: Figure S8.** Combinatorial treatment of PDX tumor explants and PD3D cell culture models improves treatment response for models resistant to monotherapy. A-D) Response evaluation of responsive (A,B) and resistant (C,D) pmCRC PDX models under combinatorial treatment of 5-FU (A,C) or trametinib (B,D) with olaparib. Explanted PDX tumor cells were treated with the indicated drug concentration and combination for 24 h in the presence of a fluorescent cytotoxicity marker. Fluorescence signals of each treatment condition (*n* = 2) indicating dead or dying cells were normalized to the respective cell confluence. Blue – decreased cytotoxicity compared to median; red – increased cytotoxicity compared to median. E) PD3D cell culture models were treated with C_max_ concentrations of 5-FU, trametinib, olaparib, or their combinations, and response was determined as cell viability after 4 days (*n* = 4).

**Additional file 9: Table S1.** pmCRC patient cohort characteristics. **Table S2**. Compound concentrations and application for preclinical treatment. **Table S3.** PDX treatment response (T/C). **Table S4.** PD3D treatment response (viability at C_max_). **Table S5.** PD3D treatment response (IC_50_). **Table S6.** Ratios and correlations of pmCRC sample types. **Table S7**. Comparison of identified CRC-related polymorphisms in the transcriptome of patient metastases and derived models. **Table S8.** In silico analysis of transcribed polymorphisms in patient metastases for prediction of therapy response. **Table S9.** Comparison of commonly mutated genes in CRC and its metastases. **Table S10.** Matched predictive biomarkers of respective drug treatment. **Table S11.** Matched predictive sequence variants for treatment response in pmCRC. **Table S12.** Validation of improved response of pmCRC models to combination therapy with PARP inhibitors.

## Data Availability

Transcriptomics and (phospho-)proteomics data have been deposited to the Gene Expression Omnibus repository (GSE180790) and to the ProteomeXchange Consortium via the PRIDE partner repository (PXD027419), respectively.

## Abbreviations

BER: Base excision repair
CMS: Consensus molecular subtype
CRC: Colorectal cancer
DsigDB: Drug signatures database
ES: Enrichment score
GSEA: Gene set enrichment analysis
H&E: Hematoxylin and eosin
MsigDB: Molecular Signatures Database
NER: Nucleotide excision repair
PD3D: Patient-derived 3D cell culture
PDX: Patient-derived xenograft
PTMsigDB: Post-translational signature database
pmCRC: Peritoneal metastasis of colorectal cancer
SoC: Standard-of-care
