# Supplementary figures and images for: Peritoneal metastasis of colorectal cancer (pmCRC): identification of predictive molecular signatures by a novel preclinical platform of matching pmCRC PDX/PD3D models
Source: Mol Cancer. 2021 Oct 21;20:129. doi: 10.1186/s12943-021-01430-7 (PMC8529724; doi:10.1186/s12943-021-01430-7)

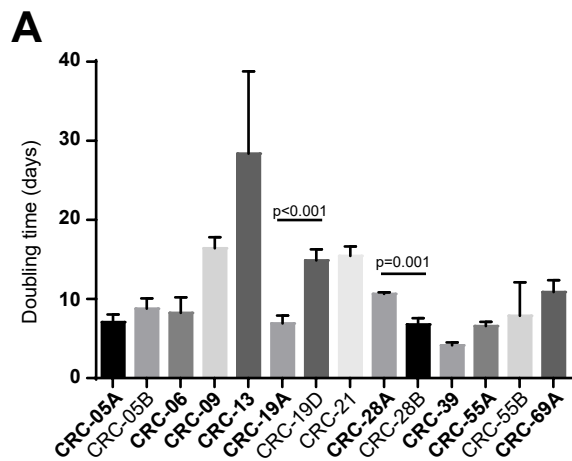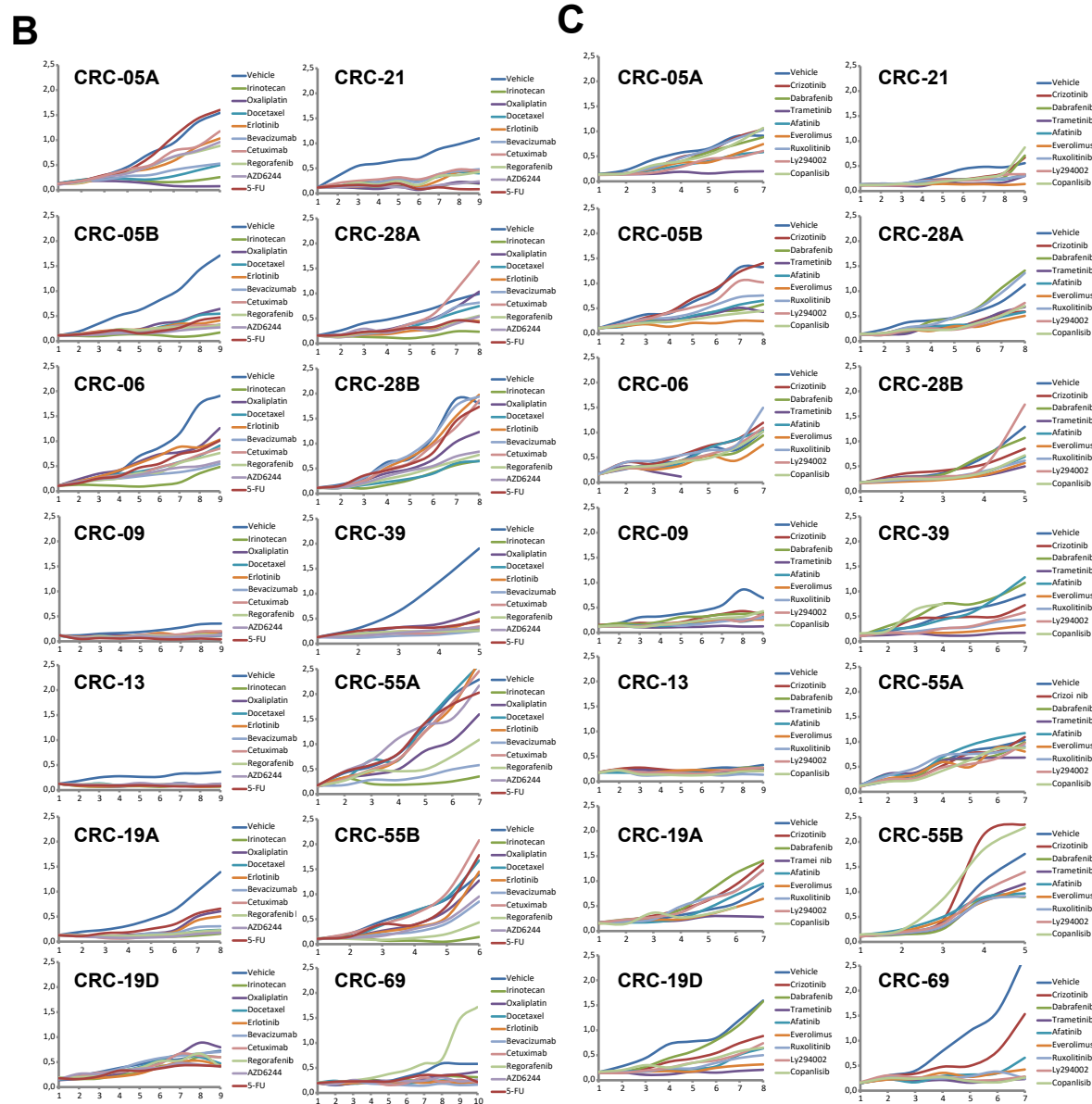

Supplementary Figure S1, Dahlmann et al., Molecular Cancer 2021

Supplement: Supplementary file 1 — Additional file 1: Figure S1. Growth and treatment response of pmCRC PDX models. A) Tumor doubling time of untreated pmCRC PDX models was assessed by volumetric measurement of the tumor growth in two dimensions with a caliper. Tumor volumes (TV) were determined by the formula: TV = (width2 x length) × 0.5, and show variances between models of the same patient, but different localization (n = 3). Bold – CRC metastasis localized at the peritoneum, regular – CRC metastasis localized at the omentum. B-C) Treatment response of pmCRC PDX models to individual SoC (B) and selected targeted drugs (C). [file 12943_2021_1430_MOESM1_ESM.pdf]

**A**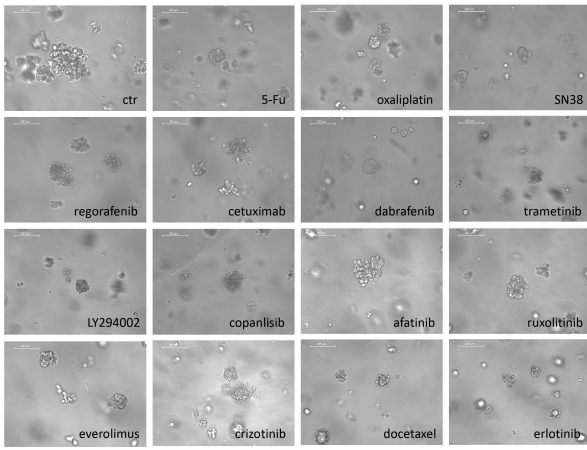**B**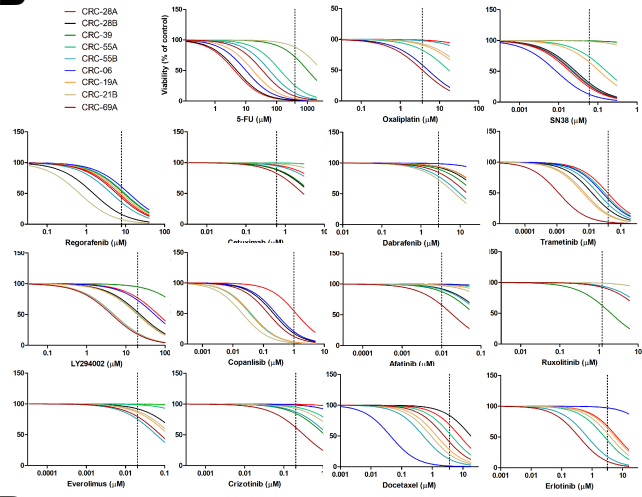**C**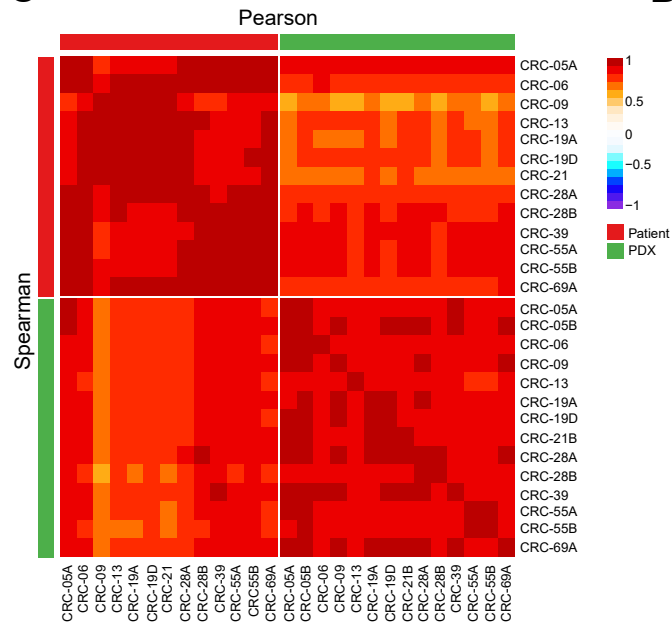**D**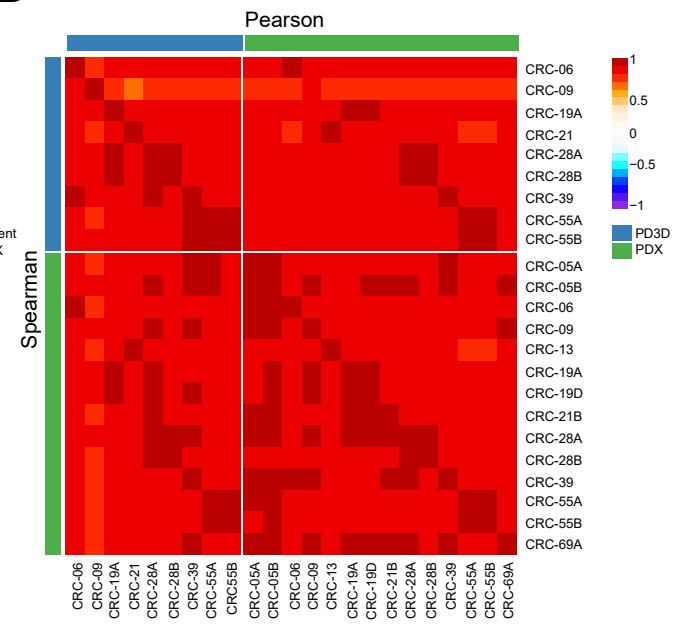**E**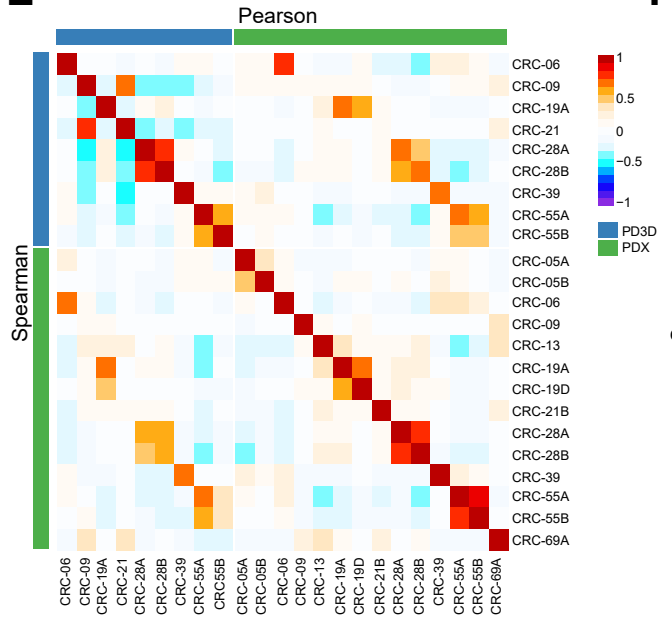**F**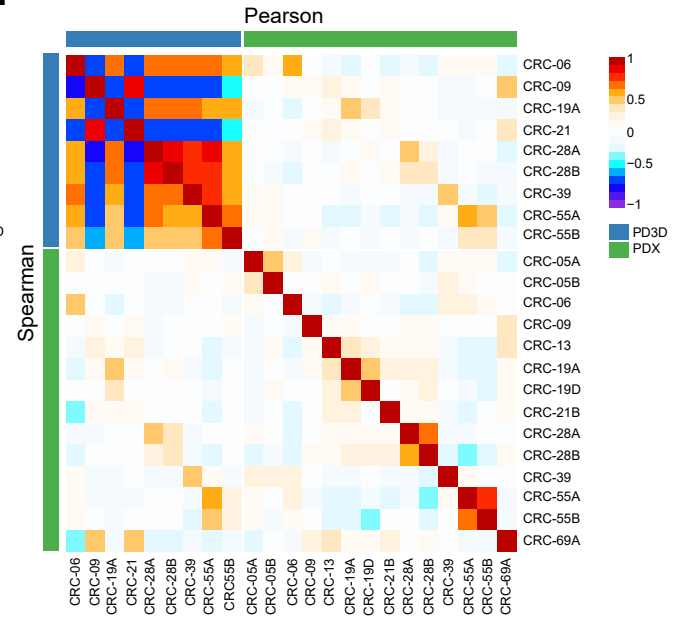

Supplement: Supplementary file 2 — Additional file 2: Figure S2. Growth and treatment response of pmCRC PD3D models and high correlation of molecular characteristics indicate high similarity of each generated pair of matched pmCRC PDX and PD3D cell culture models. A) Representative images showing the effect of the tested compounds on the size and morphology of PDX-derived PD3Ds (exemplified by CRC-21). Scale bar = 100 μm, B) Dose-response fitted curves showing cell viability after 4 days (n = 4) of the different PD3D models for each compound tested. Dotted lines show the maximum human plasma concentration of each tested drug (Cmax). C-F) Distributions of Pearson and Spearman correlation values, comparing pmCRC metastases (red bars), PDX tumors (green bars) and PD3D cell culture models (blue bars) after transcriptomic (C,D), proteomic (E) and phosphoproteomic (F) analysis. [file 12943_2021_1430_MOESM2_ESM.pdf]

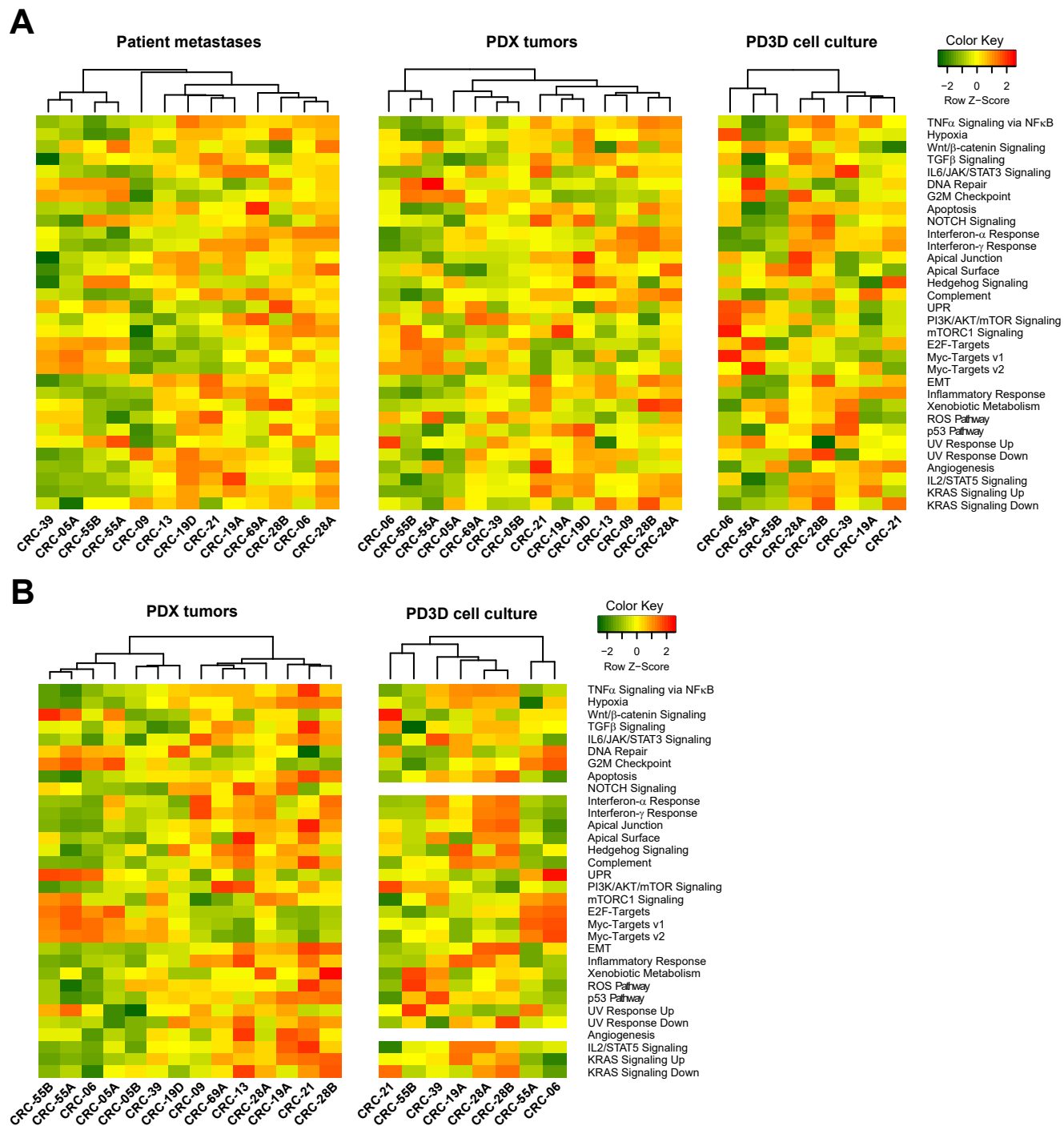

Supplementary Figure S3, Dahlmann et al., Molecular Cancer 2021

Supplement: Supplementary file 3 — Additional file 3: Figure S3. Single sample gene set analysis (ssGSEA) confirms similarity of pmCRC patient metastases and derived matched models in predicted activity of cancer-related cellular processes. A) Transcriptomic single sample enrichment analysis of cancer hallmark gene sets of pmCRC metastases, PDX tumors and PD3D culture cell models. B) Proteomic single sample enrichment analysis for cancer hallmark signatures of pmCRC PDX tumor and PD3D cell culture models. [file 12943_2021_1430_MOESM3_ESM.pdf]

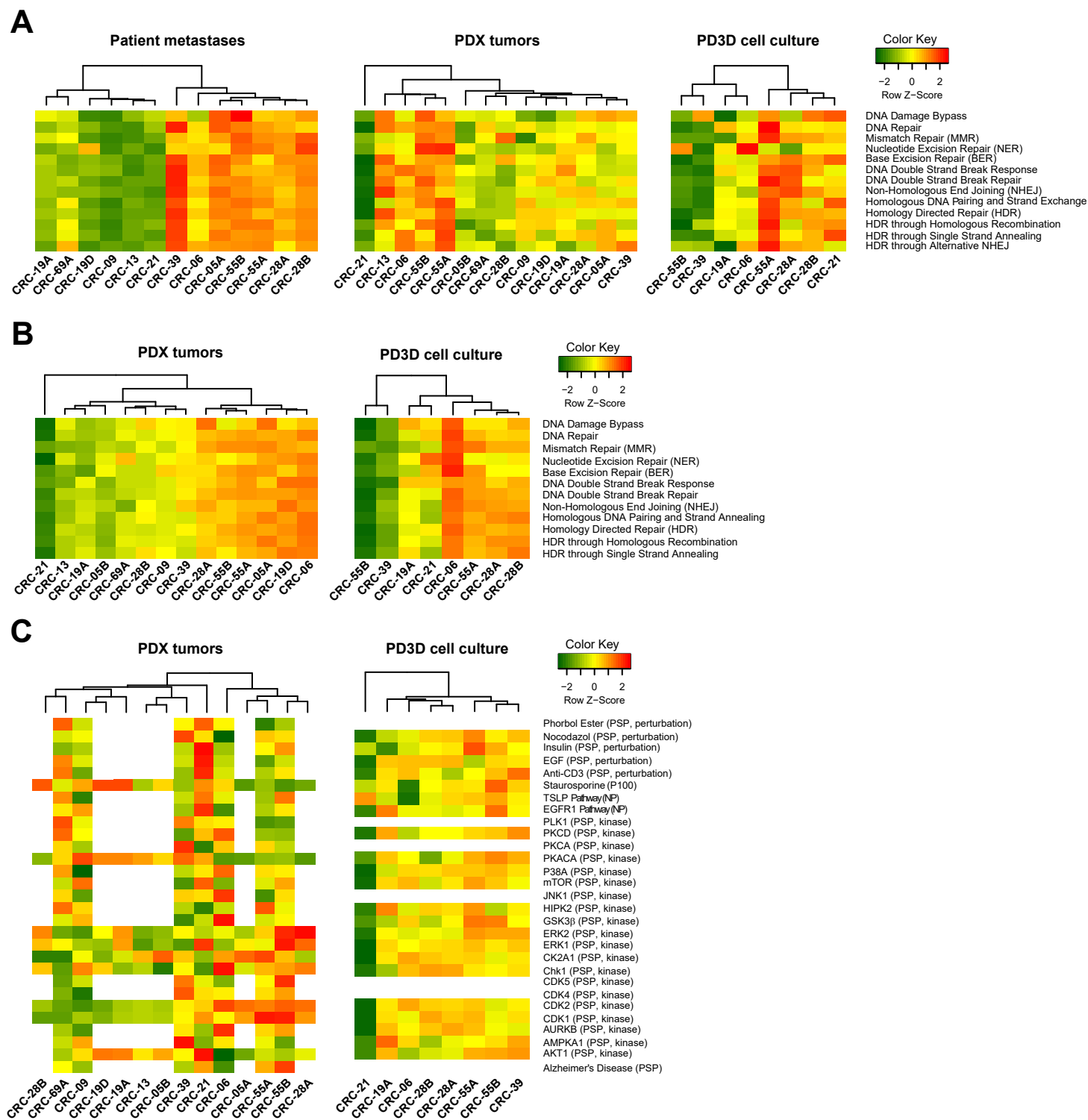

Supplementary Figure S4, Dahlmann et al., Molecular Cancer 2021

Supplement: Supplementary file 4 — Additional file 4: Figure S4. Patient samples and derived pre-clinical models are similar in their predicted activity of cellular DNA repair mechanisms and signaling pathways. A) Transcriptomic single sample enrichment analysis of reactome gene sets related to DNA repair of pmCRC metastases, PDX tumors and PD3D culture cell models. B) Proteomic single sample enrichment analysis for DNA repair signatures of pmCRC PDX tumor and PD3D cell culture models. C) Single sample enrichment analysis of phosphoproteomic signaling pathway signatures. PSP – PhosphoSitePlus, P100 – PanoramaWeb/LINCS, NP – NetPath. [file 12943_2021_1430_MOESM4_ESM.pdf]

**A**

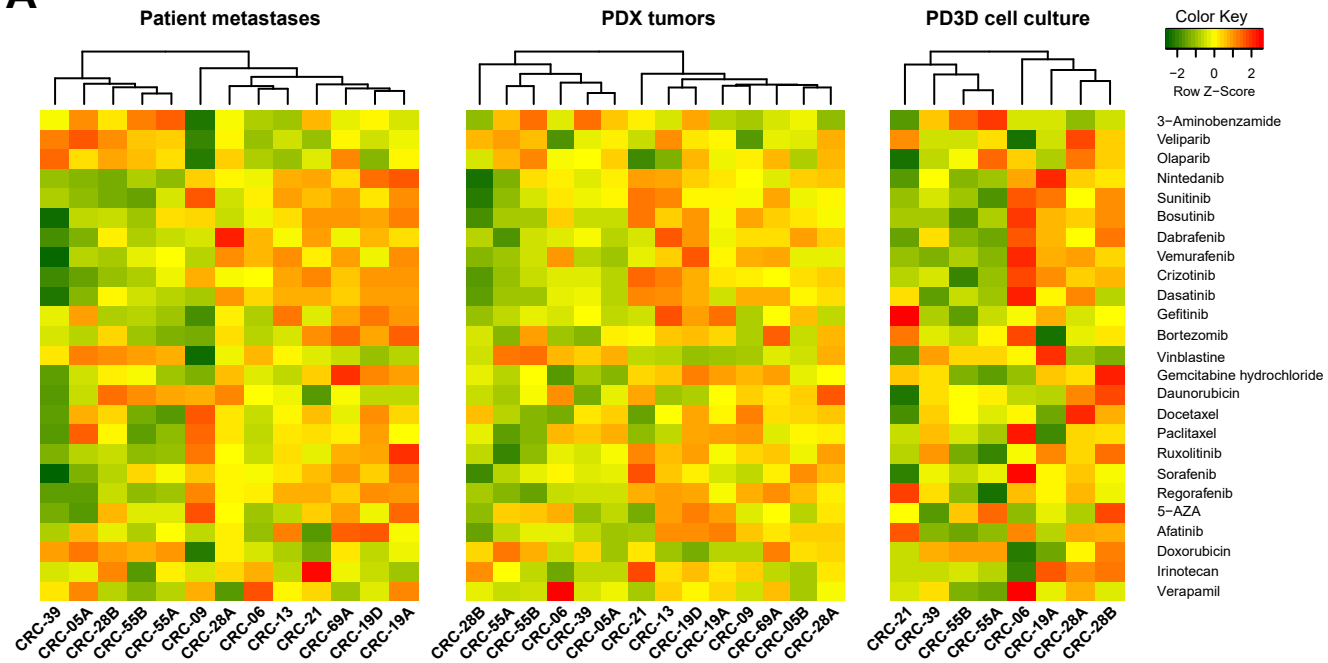

**B**

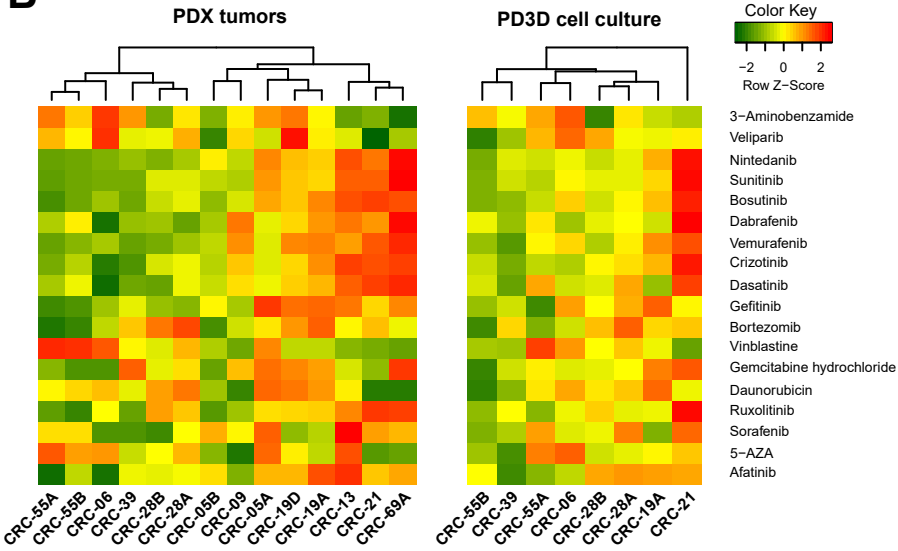

Supplementary Figure S5, Dahlmann et al., Molecular Cancer 2021

Supplement: Supplementary file 5 — Additional file 5: Figure S5. Single sample gene set analysis confirms similarity of pmCRC patient metastases and derived matched models in predicted response to anti-cancer drugs. A) Transcriptomic single sample enrichment analysis of signatures predicting treatment response to selected anti-cancer drugs of pmCRC metastases, PDX tumors and PD3D culture cell models. B) Proteomic single sample enrichment analysis for drug response signatures of pmCRC PDX tumor and PD3D cell culture models. [file 12943_2021_1430_MOESM5_ESM.pdf]

# B

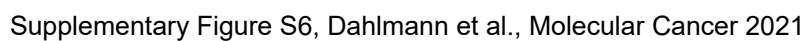

Supplement: Supplementary file 6 — Additional file 6: Figure S6. Pathway-specific gene sets reveal less active DNA damage repair pathways in pmCRC patient samples compared to preclinical models. A,B) Transcriptomic single sample enrichment analysis of pathway-specific gene sets predicting the activity of individual DNA damage repair pathways (A) and the expression distribution of pathway-specific gene expression (B). Pat – pmCRC patient metastases. [file 12943_2021_1430_MOESM6_ESM.pdf]

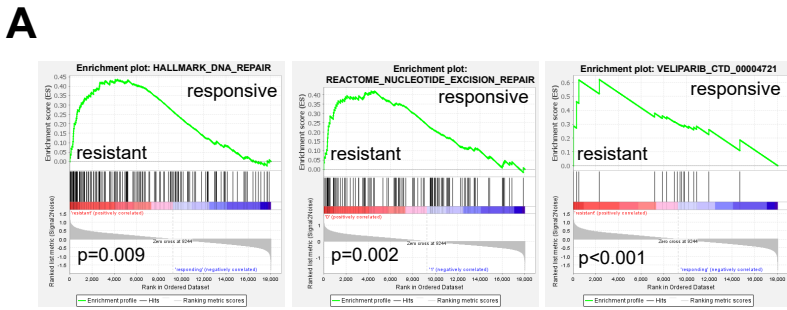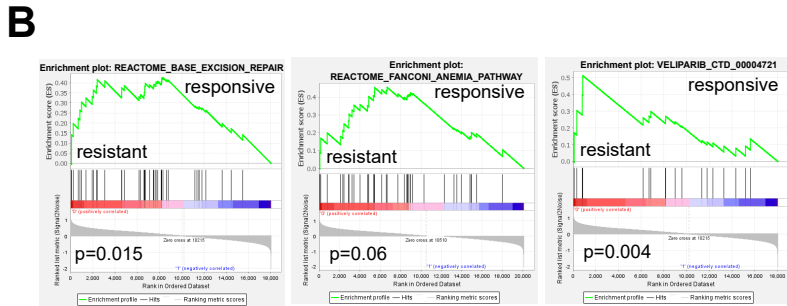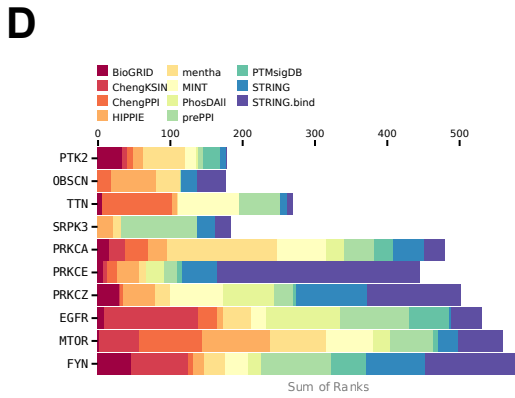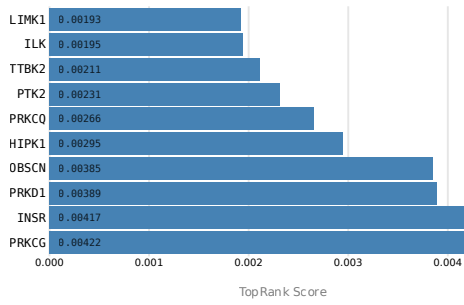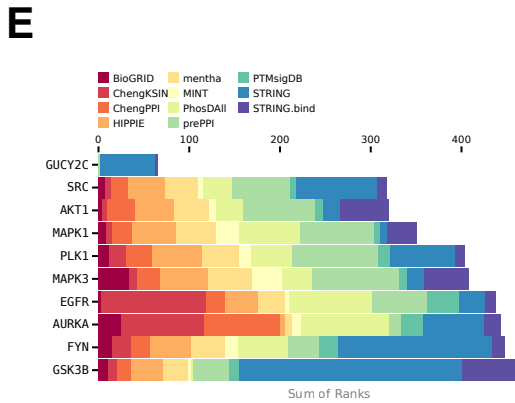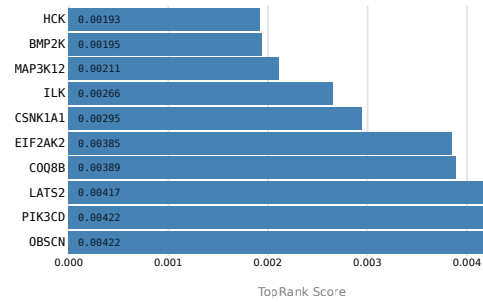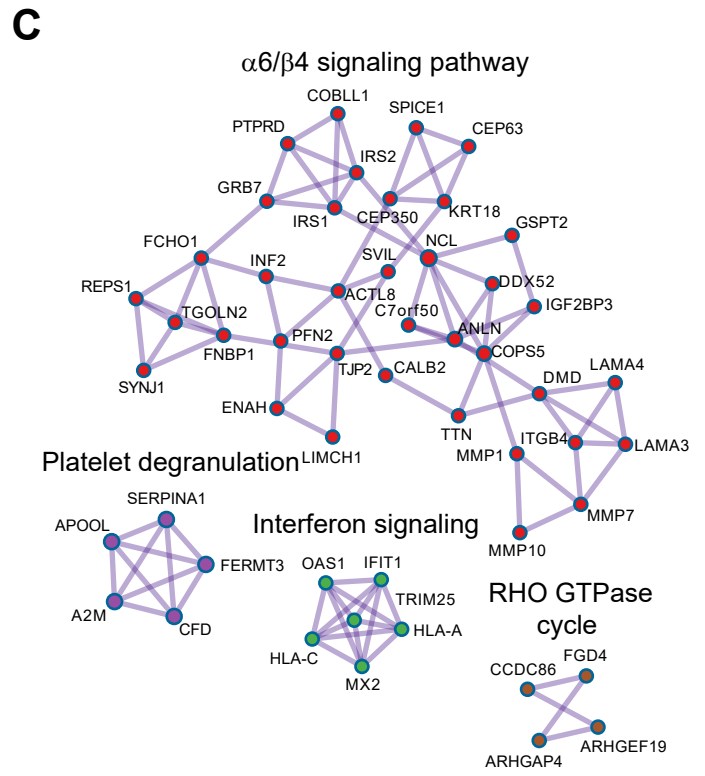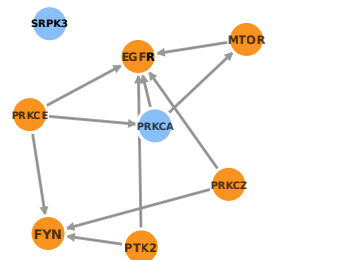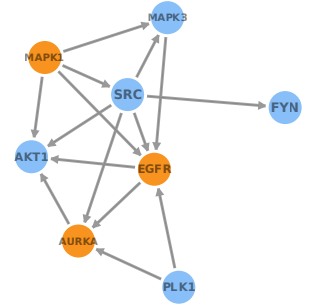

Supplementary Figure S7, Dahlmann et al., Molecular Cancer 2021

Supplement: Supplementary file 7 — Additional file 7: Figure S7. Integrated analysis of altered signatures of cellular processes, signaling pathway and kinase activity in treatment resistant and responsive PDX models. A,B) GSEA of PDX tumor transcript expression signatures grouped for response or resistance to 5-FU (A) and selumetinib (B) treatment. Significant enrichments were found for DNA repair in general (MsigDB/Hallmarks), nucleotide and base excision repair, as well as Fanconi anemia (MsigDB/Reactome) and the signature for response to veliparib treatment (DsigDB). C) Visualization of altered cellular processes (Metascape) according to integrated proteome and phosphoproteome data of 5-FU responsive and resistant PDX models. D,E) Integrated proteome and phosphoproteome data analysis (KEA3) of 5-FU resistant (D) and responsive (E) PDX models for altered kinase activity and visualization of interaction networks. Left panels list the top-10 kinases according to their sum of ranks (MeanRank score), with colors indicating the scores used from external sources. Middle panels list the top-10 kinases according to their TopRank score. Right panels visualize the interaction networks of top scoring kinases for each analysis. Blue – present in top-10 of either MeanRank or TopRank score, orange – present in both top-25 of both MeanRank and TopRank score. [file 12943_2021_1430_MOESM7_ESM.pdf]
