# Supplementary material for: Peritoneal metastasis of colorectal cancer (pmCRC): identification of predictive molecular signatures by a novel preclinical platform of matching pmCRC PDX/PD3D models
Source: Mol Cancer. 2021 Oct 21;20:129. doi: 10.1186/s12943-021-01430-7 (PMC8529724; doi:10.1186/s12943-021-01430-7)
Supplement: Supplementary file 8 — Additional file 8: Figure S8. Combinatorial treatment of PDX tumor explants and PD3D cell culture models improves treatment response for models resistant to monotherapy. A-D) Response evaluation of responsive (A,B) and resistant (C,D) pmCRC PDX models under combinatorial treatment of 5-FU (A,C) or trametinib (B,D) with olaparib. Explanted PDX tumor cells were treated with the indicated drug concentration and combination for 24 h in the presence of a fluorescent cytotoxicity marker. Fluorescence signals of each treatment condition (n = 2) indicating dead or dying cells were normalized to the respective cell confluence. Blue – decreased cytotoxicity compared to median; red – increased cytotoxicity compared to median. E) PD3D cell culture models were treated with Cmax concentrations of 5-FU, trametinib, olaparib, or their combinations, and response was determined as cell viability after 4 days (n = 4). [file 12943_2021_1430_MOESM8_ESM.pdf]

A

Olaparib [µM] × 5-FU [µM]

CRC-09	0	0.01	0.1	1	10
0	1.24E+06	1.24E+06	3.16E+06	2.37E+06	2.49E+06
2.5	2.26E+06	1.86E+06	2.08E+06	1.99E+06	6.35E+05
5	2.19E+06	1.09E+06	1.44E+06	1.88E+06	1.94E+06
10	2.22E+06	1.04E+06	1.55E+06	2.81E+06	1.71E+06
15	2.14E+06	1.90E+06	1.47E+06	2.10E+06	1.89E+06

B

Olaparib [µM] × Trametinib [µM]

CRC-05A	0	0.001	0.01	0.1	1
0	2.42E+06	4.45E+06	4.51E+06	4.53E+06	4.66E+06
2.5	4.34E+06	2.27E+06	2.59E+06	1.63E+06	1.74E+06
5	3.48E+06	2.32E+06	2.14E+06	1.58E+06	2.51E+06
10	3.11E+06	1.29E+06	2.13E+06	1.63E+06	3.27E+06
15	3.69E+06	2.21E+06	2.52E+06	4.46E+06	3.00E+06

Olaparib [µM] × Trametinib [µM]

CRC-09	0	0.001	0.01	0.1	1
0	1.04E+06	1.00E+06	2.60E+06	1.71E+06	2.93E+06
2.5	1.21E+06	6.65E+05	1.36E+06	1.57E+06	1.04E+06
5	1.38E+06	1.19E+06	1.32E+06	1.22E+06	1.35E+06
10	1.88E+06	8.79E+05	1.58E+06	1.71E+06	1.82E+06
15	2.36E+06	5.98E+05	6.70E+05	2.25E+06	1.87E+06

C

Olaparib [µM] × 5-FU [µM]

CRC-05A	0	0.01	0.1	1	10
0	3.07E+05	4.69E+05	5.72E+05	6.68E+05	6.17E+05
2.5	4.29E+05	6.38E+05	5.20E+05	6.35E+05	5.99E+05
5	6.03E+05	5.07E+05	6.37E+05	5.45E+05	5.80E+05
10	5.43E+05	6.29E+05	5.66E+05	5.54E+05	5.63E+05
15	6.86E+05	6.98E+05	4.95E+05	5.94E+05	6.12E+05

Olaparib [µM] × 5-FU [µM]

CRC-19A	0	0.01	0.1	1	10
0	1.41E+06	1.36E+06	1.80E+06	1.66E+06	1.65E+06
2.5	1.64E+06	1.48E+06	1.96E+06	1.97E+06	1.75E+06
5	1.98E+06	2.22E+06	1.69E+06	1.76E+06	1.87E+06
10	1.72E+06	1.80E+06	2.18E+06	1.47E+06	1.71E+06
15	1.64E+06	1.42E+06	1.52E+06	1.29E+06	1.31E+06

Olaparib [µM] × 5-FU [µM]

CRC-28B	0	0.01	0.1	1	10
0	6.96E+05	8.98E+05	7.67E+05	8.14E+05	1.10E+06
2.5	6.73E+05	6.92E+05	9.42E+05	8.83E+05	8.50E+05
5	8.20E+05	7.57E+05	8.79E+05	8.99E+05	8.91E+05
10	9.77E+05	7.39E+05	8.79E+05	8.74E+05	6.83E+05
15	9.51E+05	8.63E+05	7.31E+05	9.18E+05	8.79E+05

Olaparib [µM] × 5-FU [µM]

CRC-55A	0	0.01	0.1	1	10
0	2.56E+06	3.44E+06	3.22E+06	2.95E+06	2.98E+06
2.5	2.95E+06	1.73E+06	1.51E+06	1.86E+06	2.22E+06
5	2.40E+06	2.19E+06	1.69E+06	1.93E+06	1.54E+06
10	2.38E+06	2.56E+06	2.02E+06	1.83E+06	2.00E+06
15	2.64E+06	2.59E+06	2.95E+06	2.57E+06	2.11E+06

Olaparib [µM] × 5-FU [µM]

CRC-55B	0	0.01	0.1	1	10
0	2.00E+06	2.90E+06	2.80E+06	2.46E+06	2.41E+06
2.5	2.55E+06	1.83E+06	1.84E+06	2.26E+06	2.07E+06
5	2.36E+06	7.09E+05	1.05E+06	8.60E+05	1.49E+06
10	2.39E+06	6.36E+05	5.76E+05	5.28E+05	4.58E+05
15	2.83E+06	1.10E+06	1.06E+06	1.30E+06	8.52E+05

D

Olaparib [µM] × Trametinib [µM]

CRC-21	0	0.001	0.01	0.1	1
0	9.05E+04	9.45E+04	1.31E+05	1.24E+05	9.78E+04
2.5	8.47E+04	9.11E+04	9.99E+04	1.06E+05	1.18E+05
5	1.01E+05	1.14E+05	9.45E+04	1.14E+05	1.00E+05
10	8.79E+04	9.82E+04	9.46E+04	8.50E+04	1.30E+05
15	1.30E+05	1.16E+05	1.52E+05	8.37E+04	1.20E+05

Olaparib [µM] × Trametinib [µM]

CRC-55A	0	0.001	0.01	0.1	1
0	1.29E+06	1.95E+06	1.58E+06	1.64E+06	1.57E+06
2.5	1.44E+06	1.40E+06	1.07E+06	1.28E+06	1.20E+06
5	1.56E+06	1.33E+06	1.43E+06	1.25E+06	1.07E+06
10	1.24E+06	1.40E+06	1.34E+06	1.56E+06	1.43E+06
15	1.35E+06	1.61E+06	1.49E+06	1.52E+06	1.31E+06

Olaparib [µM] × Trametinib [µM]

CRC-55B	0	0.001	0.01	0.1	1
0	5.40E+05	6.72E+05	7.47E+05	7.78E+05	8.19E+05
2.5	7.76E+05	6.50E+05	5.60E+05	5.85E+05	6.31E+05
5	7.17E+05	7.29E+05	9.57E+05	6.11E+05	6.24E+05
10	7.99E+05	8.23E+05	7.95E+05	8.64E+05	8.45E+05
15	8.15E+05	8.91E+05	8.53E+05	7.44E+05	7.21E+05

Cytotoxicity

decreased — increased

E

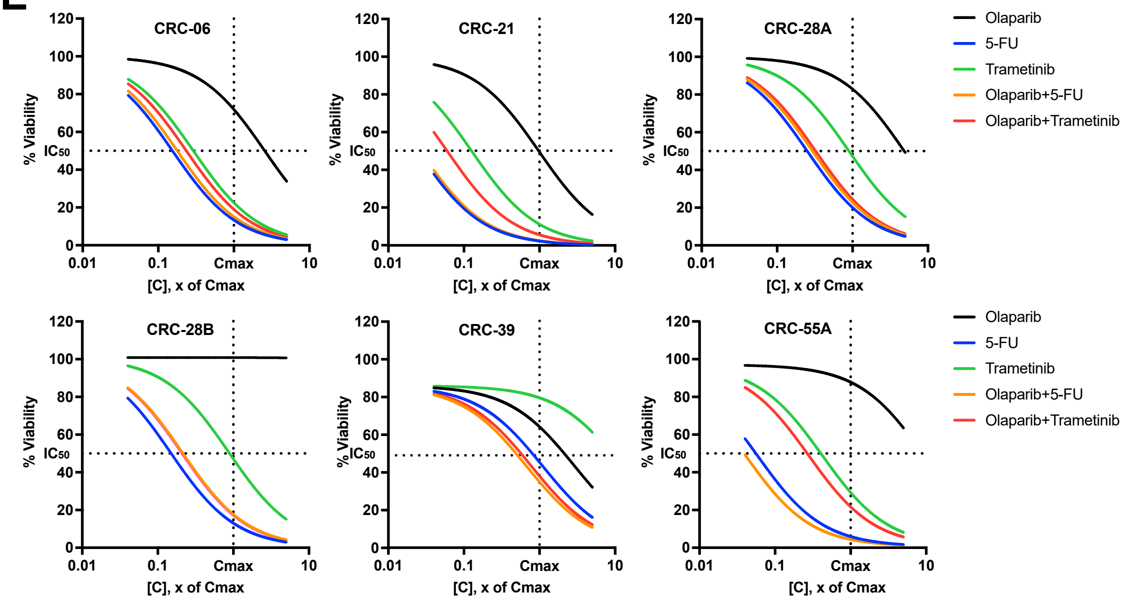

Supplementary Figure S8, Dahlmann et al., Molecular Cancer 2021
