# Supplementary material for: Peritoneal metastasis of colorectal cancer (pmCRC): identification of predictive molecular signatures by a novel preclinical platform of matching pmCRC PDX/PD3D models
Source: Mol Cancer. 2021 Oct 21;20:129. doi: 10.1186/s12943-021-01430-7 (PMC8529724; doi:10.1186/s12943-021-01430-7)
Supplement: Supplementary file 9 — Additional file 9: Table S1. pmCRC patient cohort characteristics. Table S2. Compound concentrations and application for preclinical treatment. Table S3. PDX treatment response (T/C). Table S4. PD3D treatment response (viability at Cmax). Table S5. PD3D treatment response (IC50). Table S6. Ratios and correlations of pmCRC sample types. Table S7. Comparison of identified CRC-related polymorphisms in the transcriptome of patient metastases and derived models. Table S8. In silico analysis of transcribed polymorphisms in patient metastases for prediction of therapy response. Table S9. Comparison of commonly mutated genes in CRC and its metastases. Table S10. Matched predictive biomarkers of respective drug treatment. Table S11. Matched predictive sequence variants for treatment response in pmCRC. Table S12. Validation of improved response of pmCRC models to combination therapy with PARP inhibitors. [file 12943_2021_1430_MOESM9_ESM.docx]

**Supplementary tables**

**Table S1: pmCRC patient cohort characteristics**

|  | **CRC-05A / B** | **CRC-06** | **CRC-09** | **CRC-13** | **CRC-19A / D** | **CRC-21B** | **CRC-28A / B** | **CRC-39** | **CRC-55A / B** | **CRC-69A** |
| --- | --- | --- | --- | --- | --- | --- | --- | --- | --- | --- |
| **Age** | 76 | 59 | 44 | 69 | 47 | 53 | 53 | 49 | 57 | 76 |
| **Sex** | f | m | f | m | f | m | m | m | m | f |
| **Primary colon site** | right | right | left | right | left | left | right | left | left | n.a. |
| **Pathology** | mucAC | AC | mucAC | mucAC | AC | AC | AC | AC | AC | AC |
| **Differentiation** | G2 | G3 | G2 | G2 | G2 | G3 | G2 | - | G3 | G3 |
| **UICC stage** | 4b | 4b | 4 | 4 | 4 | 3b | 4 | 2a | 4 | n.a. |
| **T/N/M stage** | 4a/2a/1b | 4b/2b/1 | 2/2b/1a | 2/0/1 | 4a/1c/1 | 4a/1a/0 | 4b/2b/1b | 3/0/0 | 4/2b/0 | 3/2b/0 |
| **Chemotherapy** | FOLFOXIRI bevac | FOLFOX pani | FOLFOXIRI cape | FOLFOX bevac | FOLFOX bevac | FOLFOXIRI pani | FOLFOX | FOLFIRI | FOLFOXIRI bevac | FOLFOX |
| **Ki-67** | n.a. | 80 % | n.a. | 60 % | n.a. | 40 % | 70 % | 60 % | 60 % | 90 % |
| **MSS/MSI** | MSS | MSS | MSS | MSS | MSS | MSS | MSS | MSS | MSS | MSS |
| **KRAS status** | mut. | w.t. | mut | mut | n.a. | w.t. | mut | mut | w.t. | mut |
| **Metastasis** | syn | syn | syn | syn | syn | meta | syn | meta | meta | meta |
| **PCI** | 15 | 9 | 16 | 39 | 13 | 14 | 21 | 2 | 20 | 4 |
| **Metastasis site** | per / om | per | per | per | per / om | om | per / om | per | per / om | per |
| **KRAS mutation** | G13D | A146T | G12D | G12C | G12D | Q61K | G12D | G12S | none | G12A |
| **CMS class** | CMS4 | CMS4 | CMS4 | CMS4 | CMS4 | CMS4 | CMS4 | CMS2 | CMS4 | CMS4 |

**f,m** – female, male; **(muc)AC** – (mucinous) adenocarcinoma; **bevac** – bevacizumab; **pani** – panitumumab; **cape** – capecitabine; **w.t.** – wild type; **syn, meta** – synchronous, metachronous; **PCI** – peritoneal cancer index; **per, om** – peritoneum, omentum; **CMS** – consensus molecular subtype

**Table S2: Compound concentrations and application for preclinical treatment**

| **Treatment** | | **PDX** | | | **PD3D** | | | |
| --- | --- | --- | --- | --- | --- | --- | --- | --- |
| **Compound** | **Target** | **dose [mg/kg]** | **application** | **sequence** | **C_max_*5 [μM]** | **C_max_ [μM]** | **C_max_/5 [μM]** | **C_max_/25 [μM]** |
| **5-FU** | DNA/RNA synth. | 100 | i.p. | q7dx4 | 2000 | 400 | 80 | 16 |
| **Oxaliplatin** | DNA/RNA synth. | 5 | i.p. | qdx5 | 18 | 3.6 | 0.72 | 0.144 |
| **Irinotecan/SN38** | Topoisomerase | 15 | i.p. | qdx5 | 0.3 | 0.06 | 0.012 | 0.0024 |
| **Regorafenib** | multi-kinase | 10 | p.o. | qd | 40 | 8 | 1.6 | 0.32 |
| **Cetuximab** | EGFR | 50 | i.p. | q7dx2 | 3 | 0.6 | 0.12 | 0.024 |
| **Dabrafenib** | BRAF | 75 | p.o. | qdx28 | 14 | 2.8 | 0.56 | 0.112 |
| **Trametinib** | MEK1/2 | 3 | p.o. | qdx28 | 0.2 | 0.04 | 0.008 | 0.0016 |
| **LY294002** | PI3K | 75 | i.p. | q4d | 100 | 20 | 4 | 0.8 |
| **Copanlisib** | PI3K | 10 | i.v. | qdx5 (5on/2off) | 5 | 1 | 0.2 | 0.04 |
| **Afatinib** | EGFR/ErbB | 10 | p.o. | qdx28 | 0.05 | 0.01 | 0.002 | 0.0004 |
| **Ruxolitinib** | JAK1/2 | 50 | p.o. | b.i.d. | 6 | 1.2 | 0.24 | 0.048 |
| **Everolimus** | mTOR | 5 | p.o. | qdx5 (5on/2off) | 0.1 | 0.02 | 0.004 | 0.0008 |
| **Crizotinib** | ALK, c-MET | 50 | p.o. | qdx28 | 1 | 0.2 | 0.04 | 0.008 |
| **Docetaxel** | microtubules | 12 | i.v. | q7dx4 | 18 | 3.6 | 0.72 | 0.144 |
| **Erlotinib** | EGFR | 50 | p.o. | qdx5 | 16 | 3.2 | 0.64 | 0.128 |
| **Bevacizumab** | VEGF | 5 | i.p. | b.i.w. | n.a. | n.a. | n.a. | n.a. |
| **Selumetinib** | MEK1/2 | 50 | i.p. | b.i.d. | n.a. | n.a. | n.a. | n.a. |
| **Olaparib** | PARP | n.a. | n.a. | n.a. | 87.34 | 17.47 | 3.49 | 0.7 |

**i.p.** – intraperitoneal; **p.o.** – per os; **i.v.** – intravenous; **qd** – daily; **q4d** – every fourth day; **q7d** – weekly; **b.i.d.** – twice a day; **b.i.w.** – twice a week; **n.a.** – not applied

**Table S3: PDX treatment response (T/C)**

|  | **CRC-05A** | **CRC-05B** | **CRC-06** | **CRC-09** | **CRC-13** | **CRC-19A** | **CRC-19D** | **CRC-21** | **CRC-28A** | **CRC-28B** | **CRC-39** | **CRC-55A** | **CRC-55B** | **CRC-69A** |
| --- | --- | --- | --- | --- | --- | --- | --- | --- | --- | --- | --- | --- | --- | --- |
| **5-FU** | - | ++ | + | ++ | ++ | + | - | +++ | + | - | ++ | - | - | + |
| **Oxaliplatin** | +++ | + | - | + | + | + | - | +++ | - | - | + | - | - | - |
| **Irinotecan** | ++ | +++ | ++ | + | ++ | ++ | - | ++ | ++ | + | ++ | ++ | +++ | - |
| **Regorafenib** | - | ++ | + | + | + | ++ | - | ++ | + | + | ++ | + | ++ | - |
| **Cetuximab** | - | ++ | + | + | + | ++ | - | + | - | - | ++ | - | - | + |
| **Dabrafenib** | - | + | - | + | - | - | - | - | + | - | - | - | - | ++ |
| **Trametinib** | ++ | + | + | ++ | - | + | ++ | + | + | + | ++ | - | - | +++ |
| **LY294002** | + | - | - | + | - | - | + | + | + | - | + | - | - | ++ |
| **Copanlisib** | - | + | - | + | - | - | + | - | - | - | - | - | - | +++ |
| **Afatinib** | - | + | - | + | - | - | + | + | - | - | - | - | - | ++ |
| **Ruxolitinib** | - | - | - | + | + | - | + | + | - | + | + | - | - | +++ |
| **Everolimus** | - | ++ | - | + | - | - | ++ | + | + | + | + | - | - | ++ |
| **Crizotinib** | - | - | - | + | - | - | - | - | - | - | - | - | - | + |
| **Docetaxel** | + | + | + | ++ | + | ++ | - | + | + | + | ++ | - | - | + |
| **Erlotinib** | - | ++ | - | + | ++ | + | - | ++ | + | - | ++ | - | - | + |
| **Bevacizumab** | + | ++ | + | + | + | ++ | - | + | + | - | ++ | + | + | + |
| **Selumetinib** | - | ++ | + | + | + | ++ | - | ++ | + | + | ++ | - | + | + |

Response categories (T/C): **+++/dark green** – 0%-10% (strong response), **++/olive** –11%-25% (moderate response), **+/yellow** – 26%-50% (minor response), **-/orange** – >50% (resistance)

**Table S4: PD3D treatment response (viability at C_max_)**

|  | **CRC-06** | **CRC-19A** | **CRC-21** | **CRC-28A** | **CRC-28B** | **CRC-39** | **CRC-55A** | **CRC-55B** | **CRC-69A** |
| --- | --- | --- | --- | --- | --- | --- | --- | --- | --- |
| **5-FU** | ++ | ++ | - | ++ | ++ | - | + | + | ++ |
| **Oxaliplatin** | + | - | - | - | - | - | + | - | + |
| **SN38** | ++ | - | - | ++ | ++ | - | - | ++ | + |
| **Regorafenib** | - | + | ++ | + | + | - | - | + | ++ |
| **Cetuximab** | - | - | - | - | - | - | - | - | - |
| **Dabrafenib** | - | - | - | - | - | - | - | - | + |
| **Trametinib** | + | ++ | ++ | + | + | - | + | + | +++ |
| **LY294002** | - | + | +++ | - | + | - | + | ++ | ++ |
| **Copanlisib** | + | +++ | +++ | + | + | - | ++ | +++ | + |
| **Afatinib** | - | - | - | - | - | - | - | - | + |
| **Ruxolitinib** | - | - | - | - | - | + | - | - | - |
| **Everolimus** | - | - | - | - | - | - | - | + | - |
| **Crizotinib** | - | - | - | - | - | - | - | - | + |
| **Docetaxel** | +++ | + | + | + | - | - | + | ++ | + |
| **Erlotinib** | - | + | + | - | + | - | + | ++ | ++ |

Response categories (viability@C_max_):**+++/dark green** – 0%-30% (strong response), **++/olive** – 31%-60% (moderate response), **+/yellow** – 61%-80% (minor response), **-/orange** – >80% (resistance)

**Table S5: PD3D treatment response (IC_50_)**

|  | **C_max_** | **CRC-06** | **CRC-19A** | **CRC-21B** | **CRC-28A** | **CRC-28B** | **CRC-39** | **CRC-55A** | **CRC-55B** | **CRC-69A** |
| --- | --- | --- | --- | --- | --- | --- | --- | --- | --- | --- |
| **5-FU** | **400** | 10.08 | 17.08 | 2960.00 | 4.30 | 5.11 | 1032.00 | 132.20 | 59.48 | 34.68 |
| **Oxaliplatin** | **3,6** | 5.09 | 36.53 | 46.04 | 301.00 | - | - | 17.07 | 161.90 | 3.65 |
| **SN38** | **0,06** | 0.01 | 0.11 | 3.85 | 0.02 | 0.03 | 10.13 | 0.17 | 0.02 | 0.02 |
| **Regorafenib** | **8** | 12.29 | 7.90 | 0.67 | 6.81 | 1.52 | 9.10 | 9.72 | 4.38 | 5.99 |
| **Cetuximab** | **0,6** | - | - | 34.32 | 14.22 | 4.58 | 5.01 | 153.20 | 10.60 | 2.84 |
| **Dabrafenib** | **2,8** | 233.10 | 44.71 | 7.49 | 46.03 | 36.90 | 24.90 | 43.23 | 10.01 | 15.49 |
| **Trametinib** | **0,04** | 0.03 | 0.01 | 0.01 | 0.04 | 0.01 | - | 0.03 | 0.02 | 0.00 |
| **LY294002** | **20** | 54.20 | 19.70 | 5.06 | 66.66 | 23.13 | 367.70 | 20.34 | 4.92 | 4.32 |
| **Copanlisib** | **1** | 0.26 | 0.04 | 0.02 | 1.20 | 0.21 | - | 0.04 | 0.04 | 0.14 |
| **Afatinib** | **0,01** | 2.90 | 0.68 | 0.37 |  | 0.12 | 0.07 | 1.28 | 0.10 | 0.02 |
| **Ruxolitinib** | **1,2** | - | - | 104.10 | - | - | 2.00 |  | 19.17 | 14.15 |
| **Everolimus** | **0,02** | - | 0.13 | 0.15 | - | 0.23 | 7.67 | 1.32 | 0.06 | 0.08 |
| **Crizotinib** | **0,2** | 11.68 | 2.48 | 2.49 | 39.58 | 11.74 | 1.11 | 3.78 | 1.38 | 0.32 |
| **Docetaxel** | **3,6** | 0.04 | 1.38 | 0.92 | 7.82 | 17.98 | - | 4.27 | 0.51 | 2.45 |
| **Erlotinib** | **3,2** | 110.70 | 5.66 | 3.99 | 6.58 | 2.57 | - | 2.58 | 0.70 | 0.37 |

IC_50_ values below C_max_ are highlighted in green

**Table S6: Ratios and correlations of pmCRC sample types**

|  | **CRC-05A** | **CRC-05B** | **CRC-06** | **CRC-09** | **CRC-13** | **CRC-19A** | **CRC-19D** | **CRC-21** | **CRC-28A** | **CRC-28B** | **CRC-39** | **CRC-55A** | **CRC-55B** | **CRC-69A** |
| --- | --- | --- | --- | --- | --- | --- | --- | --- | --- | --- | --- | --- | --- | --- |
| **RNA** |  |  |  |  |  |  |  |  |  |  |  |  |  |  |
| %PDX/Pat | 89 | n.a. | 94 | 93 | 86 | 90 | 91 | 89 | 91 | 90 | 93 | 83 | 84 | 86 |
| %PD3D/Pat | n.a. | n.a. | 91 | n.a. | n.a. | 93 | n.a. | 92 | 92 | 94 | 97 | 87 | 91 | n.a. |
| %PD3D/PDX | n.a. | n.a. | 97 | n.a. | n.a. | 100 | n.a. | 100 | 100 | 100 | 100 | 100 | 100 | n.a. |
| **PDX RNA** |  |  |  |  |  |  |  |  |  |  |  |  |  |  |
| %Human | 98 | 96 | 97 | 90 | 67 | 93 | 90 | 90 | 91 | 94 | 97 | 96 | 94 | 52 |
| **PDX Prot** |  |  |  |  |  |  |  |  |  |  |  |  |  |  |
| %Human | 78 | 79 | 83 | 79 | 48 | 63 | 73 | 63 | 84 | 78 | 81 | 83 | 79 | 36 |
| **RNA/Prot** |  |  |  |  |  |  |  |  |  |  |  |  |  |  |
| Pearson r | 0.404 | 0.669 | 0.719 | 0.689 | 0.536 | 0.669 | 0.683 | 0.623 | 0.699 | 0.556 | 0.708 | 0.723 | 0.718 | 0.375 |
| **PD3D RNA** |  |  |  |  |  |  |  |  |  |  |  |  |  |  |
| %Human | n.a. | n.a. | 100 | n.a. | n.a. | 100 | n.a. | 83 | 100 | 99 | 100 | 100 | 100 | n.a. |
| **PD3D Prot** |  |  |  |  |  |  |  |  |  |  |  |  |  |  |
| %Human | n.a. | n.a. | 97 | n.a. | n.a. | 97 | n.a. | 2^*^ | 96 | 96 | 96 | 97 | 79 | n.a. |
| **RNA/Prot** |  |  |  |  |  |  |  |  |  |  |  |  |  |  |
| Pearson r | n.a. | n.a. | 0.681 | n.a. | n.a. | 0.623 | n.a. | 0.016^*^ | 0.613 | 0.666 | 0.648 | 0.649 | 0.585 | n.a. |

**RNA** – transcripts after mRNA sequencing; **Prot** – proteins after MS proteomics; **^*^** - matrigel contamination

**Table S7: Comparison of identified CRC-related polymorphisms in the transcriptome of patient metastases and derived models**

|  | | ***APC*** | ***AXIN2*** | ***TP53*** | ***SMAD*** | ***KRAS*** | ***EGFR*** | ***ERBB2*** | ***ERBB3*** | ***FGFR4*** | ***MAPK3*** | ***PIK3CA*** | ***BRCA2*** | ***FBXW7*** | ***KMT2D*** | ***ATM*** | ***RNF43*** | ***NOTCH1*** | ***NOTCH3*** | ***GNAS*** | ***EP300*** |
| --- | --- | --- | --- | --- | --- | --- | --- | --- | --- | --- | --- | --- | --- | --- | --- | --- | --- | --- | --- | --- | --- |
| **CRC-05A** | **P** | **R1450*** V1822D |  | P72R **R175H** | C499R **L533R** | **G13D** |  | I655V | G284R S1234F | V10I P136L |  |  | **T2125fs** V2466A |  |  |  | I47V L418M |  | A2223V |  |  |
|  | **X** | **R1450*** V1822D |  | P72R **R175H** | **L533R** | **G13D** |  | I655V | G284R S1234F | V10I P136L |  |  | S131fs S384F **N863fs** **N1784fs** V2466A **E2981fs** **T3033fs** |  |  | **E2304fs** | I47V L418M |  | A2223V |  |  |
| **CRC-05B** | **X** | **R1450*** **T1556fs** V1822D |  | P72R **R175H** | **L533R** | **G13D** | L480fs | I655V P1170A | G284R  S413fs S1234F | V10I P136L |  |  | S384F **I605fs** **N863fs** **N986fs** V2466A **T3033fs** |  | A3552fs | **E2304fs** | I47V L418M |  |  |  |  |
| **CRC-06** | **P** | **Y953*** V1822D | P50S | P72R | **D52fs** | A146T | T477* | P1170A |  | P136L G388R |  |  | Y2884F **T3033fs** |  | R1918H G2493E |  | I47V L418M |  | V1952M A2223V |  |  |
|  | **X** | **Y953*** P1549L V1822D | P50S | P72R |  | A146T | L480fs | P1170A |  | P136L G388R |  |  | V2466A Y2884F **T3033fs** |  | R1687fs R1918H G2493E |  | I47V L418M |  |  |  |  |
|  | **3** | **Y953*** V1822D | P50S | P72R |  |  |  | P1170A |  | P136L G388R |  |  | V2466A Y2884F |  | R1918H G2493E | **S214fs** | I47V L418M |  | V1952M A2223V |  |  |
| **CRC-09** | **P** | V1822D K2051fs | P50S | P72R | **D52fs** |  | R521K | P1170A |  |  |  | R38H | V2466A |  |  | D1853N | L418M |  | A2223V |  |  |
|  | **X** | I495V P1549L V1822D | P50S | P72R F134C |  | **G12D** | A202G R521K I1165M |  |  |  |  | R38H | **N986fs** K1690N V2466A |  |  | I1581fs **L1814fs** | I47V L418M |  | A2223V |  |  |
| **CRC-13** | **P** | V1822D | P50S | P72R |  | **G12C** | R521K | I655V P1170A |  | V10I P136L |  |  | **I605fs** **N1766fs** V2466A **T3033fs** E3316fs |  |  |  |  | R1279H | A2223V |  | I997V **N1236fs** |
|  | **X** | P429Q |  | Q165K | I347V | **G12C** | R521K | F55Y I655V P1170A |  | V10I P136L | S58fs |  |  |  |  |  |  | R1279H | A2223V |  | N251S P261S I997V |
| **CRC-19A** | **P** | V1822D | P50S N412S | **R282W** | **D52fs** | **G12D** |  | P1170A | S413fs |  | E323K |  | L1390fs V2466A |  | P813L |  | I47V **R113*** |  | A2223V |  |  |
|  | **X** | K1543E V1822D | P50S | **R282W** | **G352E** | **G12D** |  | P8T P1170A |  |  | E323K |  | T1692fs V2466A |  | P813L |  | I47V **R113*** |  |  |  |  |
|  | **3** | V1822D | P50S | **R282W** | **G352E** | **G12D** |  | P8T P1170A |  |  | E323K |  | **T3033fs** V2466A |  | P813L |  | I47V **R113*** |  |  |  |  |
| **CRC-19D** | **P** | **T1556fs** V1822D | P50S N412S | **R282W** | **G352E** | **G12D** |  | P1170A |  |  | E323K |  | V2466A |  | P813L |  | I47V **R113*** |  | A2223V |  |  |
|  | **X** | A59T P429Q V1822D | P50S | **R282W** | **G352E** | **G12D** |  | P1170A |  |  | E323K |  | V2466A |  | P813L | **I1581fs** | I47V **R113*** |  |  |  |  |
| **CRC-21** | **P** | E19Q | P50S | P72R |  | **Q61K** | R521K | I655V |  | P136L A174T |  |  | N372H |  |  | S49C |  |  | A2223V |  |  |
|  | **X** | E19Q P429Q K1543E P1549L V1822D | P50S |  |  | **Q61K** | R521K | I655V |  | P136L |  |  | N372H **N1784fs** V2466A **E2981fs** **T3033fs** |  |  | S49C |  |  |  |  |  |
|  | **P3D** |  | P50S | Q165K |  | **G12C** | R521K | I655V P1170A |  | V10I P136L |  |  | V2466A |  |  |  |  | R1279H | A2223V |  |  |
| **CRC-28A** | **P** | I495V T838M **R1450*** V1822D | P50S S762N | P72R **R175H** |  | **G12D** | R521K | I655V P1170A |  | P136L G388R |  |  | V2466A Y3098H |  |  | **L1176fs** | I47V L418M |  | A2223V |  | I997V |
|  | **X** | **R805*** **R1450*** V1822D | P50S S762N | P72R **R175H** | K428E | **G12D** | L480fs R521K | I655V P1170A |  | P136L G388R |  |  | V2466A **T3033fs** Y3098H | R479Q |  |  | I47V L418M |  | A2223V |  | I997V |
|  | **P3D** | **R805*** **R1450*** V1822D | P50S S762N | P72R **R175H** |  | **G12D** | R521K | I655V P1170A |  | P136L G388R |  |  | N372H V2466A Y3098H | R479Q |  |  | I47V L418M |  | A2223V |  |  |
| **CRC-28B** | **P** | **R805*** **R1450*** V1822D | P50S S762N | P72R **R175H** |  | **G12D** | R521K | P8T I655V P1170A |  | P136L G388R |  |  | V2466A Y3098H |  |  |  | I47V L418M |  | A2223V |  | I997V |
|  | **X** | **R1450*** | P50S S762N | P72R **R175H** | K428E | **G12D** | R521K | I655V P1170A |  | P136L G388R |  |  | **L3045fs** | R479Q |  |  | I47V L418M |  | A2223V |  |  |
|  | **3** | **R805*** **R1450*** V1822D | P50S S762N | P72R **R175H** |  | **G12D** | R521K | I655V P1170A | S413fs | P136L G388R |  |  | N372H V2466A Y3098H | R479Q |  |  | I47V L418M |  | A2223V |  |  |
| **CRC-39** | **P** | **E1353*** V1822D |  | P72R **E258K** |  | **G12S** |  | P1170A | S413fs S1119C | P136L G388R |  |  | V2466A **T3033fs** |  |  |  | I47V L418M |  | H1133Q A2223V |  |  |
|  | **X** | I495V I983V **E1353*** S1620A V1822D |  | P72R **E258K** |  | **G12S** |  | P1170A | S1119C | P136L G388R |  |  | V2466A **T3033fs** |  | R1687fs |  | I47V L418M |  |  |  |  |
|  | **P3D** | **E1353*** V1822D |  | P72R **E258K** |  | **G12S** |  | P1170A | S1119C | P136L G388R |  |  | V2466A |  |  |  | I47V L418M |  | A2223V |  |  |
| **CRC-55A** | **P** | E1317Q **T1556fs** |  | P72R **P151S** | **D52fs** |  | R521K | P8T P1170A |  | P136L G388R |  |  | N372H V2466A **T3033fs** |  | R1687fs | **S214fs** F858L P1054R | I47V L418M | E848K | A2223V |  |  |
|  | **X** | V1822D |  | P72R **P151S** |  |  | R521K | P8T P1170A |  | P136L D184G G388R |  |  | V2466A **T3033fs** |  |  |  | I47V L418M | E848K |  |  |  |
|  | **3** | E1317Q **T1556fs** V1822D |  | P72R **P151S** A189V Q634H |  |  | R521K | P8T P1170A |  | P136L |  |  | N372H G1529E V2466A |  |  | F858L | I47V L418M | E848K |  |  |  |
| **CRC-55B** | **P** | V1822D |  | P72R **P151S** |  |  | L480fs R521K | P8T P1170A | S413fs | P136L G388R |  |  | **V726fs** V2466A **T3033fs** |  |  | F858L | I47V L418M | E848K | A2223V |  |  |
|  | **X** | M701I **T1556fs** V1822D |  | P72R **P151S** |  |  | R521K | P8T P1170A |  | P136L G388R |  |  |  |  |  |  | I47V L418M | E848K |  |  |  |
|  | **3** | E1317Q **T1556fs** V1822D K1156R |  | P72R **P151S** A189V Q634H |  |  | R521K | P8T P1170A |  | P136L G388R |  |  | V2466A |  |  | F858L | I47V L418M | E848K |  |  |  |
| **CRC-69A** | **P** | P429Q **L1302fs** V1822D | P50S | P72R | **D52fs** | **G12A** | L480fs | I655V P1170A |  | P136L | E323K | I391M | V2466A **E2981fs** **T3033fs** |  |  |  |  |  | A2223V | **R844C** | M289V |
|  | **X** | A59T A61T S296G I495V R1171H P1188S **L1302fs** K1543E P1549L V1822D A2650P | P50S | P72R |  | **G12A** | I966fs | I655V E967fs A1181T S1190P |  | P136L | M30V S58fs R67S E323K | I347V M1040T M1040I | V2466A |  |  | **E2304fs** F2799I |  |  |  | **R844C** | P261S M289V N301S F1487L |

**P** – patient metastasis; **X** – PDX model; **3** – PD3D model; **bold** – pathogenic (ClinVar)

**Table S8: *In silico* analysis of transcribed polymorphisms in patient metastases for prediction of therapy response**

| **Therapy** | **Gene** | **CRC-05** | **CRC-06** | **CRC-09** | **CRC-13** | **CRC-19** | **CRC-21** | **CRC-28** | **CRC-39** | **CRC-55** | **CRC-69** |
| --- | --- | --- | --- | --- | --- | --- | --- | --- | --- | --- | --- |
| **WNTi** | *APC* | E761* **R1450*** **T1556fs** V1822D K2051fs | **Y935*** V1822D | K2051fs V1822D | V1822D | **T1556fs** R1788fs V1822D K2051fs | E19Q | **R805* R1450*** P1594fs V1822D K2051fs | **E1353*** V1822D K2051fs | E1317Q **T1556fs** P1594fs V1822D | P429Q **L1302fs** V1822D |
|  | *RSPO2* |  |  | L186P |  |  |  |  |  |  |  |
| **PORCUPINEi** | *RNF43* | I47V R343H L418M | I47V R343H L418M | L418M | P231L | I47V **R113*** | R117H R337* | I47V L418M | I47V P231L L418M | I47V L418M | R117H P686R |
|  | *ZNRF3* |  |  | A725fs |  |  | S557delta |  |  |  |  |
| **ERBBi** | *EGFR* |  | T477* | R521K | R521K |  | R521K | R521K | R521K | L480fs | L480fs |
|  | *ERBB2* | I655V | P1170A | P1170A | I655V P1170A | P1170A | I655V | P8T I655V P1170A | P1170A | P8T P1170A | I655V P1170A |
|  | *ERBB3* | G284R S1234F |  |  |  | S413fs |  |  | S413fs S1119C | S413fs |  |
|  | *ERBB4* |  |  |  | S418fs |  |  |  |  |  |  |
|  | *ERRFI1* | S273F |  |  | R247K |  |  |  |  |  |  |
| **FGFRi** | *FGFR2* |  |  |  |  |  |  |  | I654fs |  |  |
| **KDRi** | *KDR* | N511fs G539R | Q472H | Q472H | Q472H | V297I Q472H N511fs |  | Q472H |  | Q472H |  |
| **Sorafenib** | *RET* |  |  |  | W543* |  |  |  |  |  | R897L |
| **Sunitinib** | *FLT3* |  |  |  | T227M |  | T227M | R311W |  | T227M | T227M |
| **JAKi** | *JAK2* |  |  | N457fs |  |  |  |  |  |  |  |
|  | *GNAS* |  |  |  |  |  |  | R99K K100E |  |  | **R844C** |
| **Imatinib** | *PDGFRA* |  |  |  |  | S478P |  |  |  |  |  |
|  | *PDGFRB* | E485K |  |  |  |  |  |  |  |  | E485K |
|  | *KIT* | V409* |  | M541L |  | K236fs |  | Y846fs |  |  |  |
|  | *ABL1* |  |  |  |  |  |  |  | S972L |  |  |
| **RAFi** | *MITF* |  |  |  |  |  |  |  |  | E291D E291V |  |
|  | *NRAS* |  |  |  |  | L171fs |  |  |  |  |  |
| **MEKi** | *KRAS* | **G13A** | A146T |  | **G12C** | **G12D** | **Q61L** | **G12D** | **G12S** | C186fs | **G12A** |
| **PI3K/AKTi** | *PIK3CA* |  |  | R38H |  |  |  | C604R H1065Y |  | L99fs | L99fs I391M |
|  | *PIK3R1* |  |  |  |  | S460fs | M326I |  |  | I82fs |  |
| **MTORi** | *MTOR* |  |  |  |  |  |  | R624H |  |  |  |
|  | *TSC1* |  | M322T | M322T | M322T | **N891fs** | M322T | V178I |  | **N891fs** | L627V |
|  | *TSC2* |  |  |  |  |  | **V299fs** R367Q | **V299fs** L826M | **V299fs** | **V299fs** | R1369T |
| **Crizotinib** | *ALK* |  |  | I1461V |  | I1461V | I1461V | Y1239C | P1370L |  |  |
|  | *ROS1* |  |  |  |  |  |  |  |  |  | S1109L |
| **Cisplatin** | *ERCC2* | D312N | D312N K751Q | A182V D312N K751Q | D312N K751Q |  | D312N K751Q |  | D312N K751Q | D312N K751Q |  |
| **AURKAi** | *AURKA* | F31I P57V M373V | F31I | P57V | F31I P57V | M373V | P57V | F31I P57V | P57V | P57V | P57V |
| **WEE1i / CHK1i** | *TP53* | P72R **R175H** | P72R S94* | P72R | P72R | **R282W** | P72R | P72R **R175H** | P72R **E258K** | P72R **P151S** | P72R |
| **MDM4i** | *MDM4* |  |  |  |  |  |  | L203fs |  |  |  |
| **PARPi** | *BRCA1* | S1613G | P871L E1038G K1183R S1613G |  |  |  |  | P871L E1038G K1183R S1613G | P871L | P871L E1038G K1183R S1613G | D693N P871L E1038G K1183R S1613G |
|  | *BRCA2* | **T2125fs** V2466A | Y2884F **T3033fs** | V2466A | **I605fs** **N1766fs** V2466A **T3033fs** E3316fs | L1390fs V2466A | N372H | N854fs V2466A Y3098H | F1216fs **T3033fs** | N372H **V726fs** V2466A **T2607fs T3033fs** | V2466A **E2981fs T3033fs** |
|  | *ATM* |  |  | D1853N | I2233T | N619fs | S49C | **L1176fs** | E594A E594fs | **S214fs** F858L P1054R |  |
|  | *ATR* | R1814fs | M211T S927fs R1814fs H2437Y H2437L | M211T F929L R1814fs | M211T S271fs | R1814fs | M211T R2425Q H2437Y H2437L E2438V E2438D E2438* T2439L T2439C | M211T S271fs | M211T R1814fs R2425Q | M211T S271fs V316I V959M | M211T R1814fs |
|  | *TMPRSS2* | V160M | V160M |  |  | V160M |  | V160M |  | V160M | V160M |
|  | *CDK12* | S133fs | S133fs | S133fs | S133fs | S133fs |  | S133fs | S133fs | S133fs | S133fs |
| **IFG1-Ri** | *IGFR1* |  |  |  |  | C230Y C231S C231Y H232Y | S24fs |  |  |  |  |
| **NOTCHi** | *NOTCH1* |  |  |  | R1279H |  |  | N2248K |  | E848K |  |
|  | *NOTCH2* |  |  |  | D1327G |  |  |  |  |  |  |
| **HHi** | *SMO* |  |  |  |  |  |  |  |  | G29R |  |
|  | *PTCH1* | P1315L | P1315L |  |  |  |  | P1315L | P1315L | E53K P1315L |  |
| **HDACi** | *KTM2A* | A930fs I3105fs | I3105fs | I3105fs | I3105fs | G156fs I3105fs | A930fs G1746E I3105fs | A383fs K929fs P3668fs I3105fs | A930fs S1325N | I256fs A383fs I3105fs | A383fs I3105fs |
|  | *BAP1* | G624R |  |  |  |  |  |  |  |  |  |
|  | *BRD3* |  | K435Q |  |  |  | K435Q |  |  |  |  |
|  | *BRD4* |  |  |  |  |  |  |  |  | R12K H1145Y |  |
| **IDHi** | *IDH1* | V178I |  |  | V178I |  |  |  |  |  |  |
| **EPHi** | *EPHA3* | S548fs |  | R580fs | R580fs | P845H | K142fs T924R |  |  | R580fs R914H T924R | T924R |
| **EZH2i** | *EZH2* | D185H |  |  |  |  |  |  |  |  |  |
| **Enzalutamide** | *AR* |  |  | **N849fs** |  |  |  |  |  |  |  |
| **CDKi** | *CDKN1A* |  |  |  |  |  |  | S31R |  | S31R |  |
|  | *CDKN1B* | V109G |  |  | V109G | V109G |  |  |  | V109G | V109G |
|  | *CDKN2A* |  | A148T |  |  |  |  | A148T | A148T |  |  |
|  | *CDK6* |  |  |  |  |  |  | D110N |  |  |  |
|  | *CCND3* | S259A | S259A | S259A | E253D S259A | S259A | S259A | S259A | S259A | S259A | S259A |

***i** – inhibitor; **bold** – pathogenic (ClinVar)

**Table S9: Comparison of commonly mutated genes in CRC and its metastases**

| **Gene** | **Yaeger *et al.* (11)**  **(m)CRC [pT+M]** | **El-Deiry *et al.* (12)**  **(m)CRC [pT]** | **this report**  **pmCRC[M]** |
| --- | --- | --- | --- |
| *APC* | 79% | 67.9% | 57% |
| *TP53* | 78% | 58.3% | 57% |
| *KRAS* | 44% | 44.0% | 64% |
| *PTEN* | 20% | 2–5% | 0% |
| *PIK3CA* | 18% | 14.1% | 7% |
| *BRAF* | 16% | 9.60% | 0% |
| *SMAD4* | 16% | 12.9% | 50% |
| *BRCA2* | 16% | n.p. | 85% |
| *PIK3R1* | 14% | n.p. | 0% |
| *NF1* | 13% | n.p. | 0% |
| *ERBB3* | 10% | n.p. | 7% |
| *RNF43* | 9% | n.p. | 14% |
| *CTNNB1* | 8% | 1–2% | 0% |
| *ERBB2* | 8% | 1–2% | 0% |
| *BRCA1* | 8% | n.p. | 0% |
| *FBXW7* | n.p. | 7.10% | 0% |
| *TSC1* | 4% | n.p. | 7% |
| *AKT1* | 3% | 1–2% | 0% |
| *EGFR* | 2% | 1–2% | 0% |
| *GNAS* | n.p. | 2–5% | 7% |
| *ATM* | n.p. | 2–5% | 14% |
| *MAP2K1* | 2% | n.p. | 0% |
| *TSC2* | 1% | n.p. | 29% |
| *NTRK2* | 1% | n.p. | 0% |
| *MET* | 1% | n.p. | 0% |
| *MTOR* | 1% | n.p. | 0% |
| *RAF1* | 1% | n.p. | 0% |
| *HRAS* | 1% | n.p. | 0% |

**(m)CRC** – metastasized and non-metastasized colorectal cancer;

**pT** – primary tumors ; **M** – metastases; **n.p.** – data not published

**Table S10: Matched predictive biomarkers of respective drug treatment**

| **5-FU** | | | | | | | | |
| --- | --- | --- | --- | --- | --- | --- | --- | --- |
| Gene | log2.Pat | log2.PDX | Sens.Pat | Spec.Pat | Sens.PDX | Spec.PDX | Prediction | Description |
| *PCDHGB2* | 1.9 | 4.8 | 100% | 78% | 75% | 100% | Response | Protocatherin gamma-B2 |
| *EPOP* | -1.4 | -1.8 | 100% | 100% | 78% | 100% | Resistance | Elongin BC and Polycomb repressive complex 2-associated protein |
| *ATP6V1C2* | -2.3 | -1.7 | 89% | 100% | 89% | 100% | Resistance | V-type proton ATPase subunit C2 |
| *SLC7A5* | -2.7 | -2.2 | 89% | 100% | 89% | 100% | Resistance | Large neutral amino acids transporter small subunit |
| *SFTA2* | -7.4 | -3.3 | 100% | 100% | 78% | 100% | Resistance | Surfactant-associated protein-2 |
|  |  |  |  |  |  |  |  |  |
| **Trametinib** | | | | | | | | |
| Gene | log2.Pat | log2.PDX | Sens.Pat | Spec.Pat | Sens.PDX | Spec.PDX | Prediction | Description |
| *CYP4X1* | 2.3 | 5.6 | 100% | 88% | 100% | 88% | Response | Cytochrome P450 4X1 |
| *ERP27* | -1.4 | -3.0 | 75% | 100% | 75% | 100% | Resistance | Endoplasmic reticulum resident protein 27 |
| *H2AFY2* | -1.9 | -6.0 | 88% | 100% | 75% | 100% | Resistance | Core histone macro-H2A.2 |
| *ADGRF4* | -2.1 | -1.7 | 88% | 100% | 100% | 100% | Resistance | Adhesion G protein-coupled receptor F4 |
| *BAMBI* | -2.3 | -1.5 | 75% | 100% | 88% | 80% | Resistance | BMP and activin membane-bound inhibitor homolog |
| *FOXH1* | -4.2 | -2.8 | 75% | 100% | 50% | 100% | Resistance | Forkhead box protein H1 |
| *IL36RN* | -5.6 | -2.5 | 75% | 100% | 75% | 100% | Resistance | Interleukin-36 receptor antagonist protein |
| *SLC30A10* | -5.7 | -5.1 | 75% | 100% | 75% | 100% | Resistance | Zinc transporter 10 |
| *TMPRSS11E* | -6.8 | -4.5 | 88% | 100% | 75% | 100% | Resistance | Transmembrane protease serine 11E |
|  |  |  |  |  |  |  |  |  |
| **Erlotinib** | | | | | | | | |
| Gene | log2.Pat | log2.PDX | Sens.Pat | Spec.Pat | Sens.PDX | Spec.PDX | Prediction | Description |
| *CITED4* | -1.8 | -2.6 | 100% | 100% | 100% | 100% | Resistance | Cbp/p300-interacting transactivator 4 |
| *ATP6V1C2* | -2.3 | -2.9 | 80% | 100% | 90% | 100% | Resistance | V-type proton ATPase subunit C2 |
| *BMP7* | -3.2 | -4.3 | 90% | 100% | 90% | 100% | Resistance | Bone morphogenic protein 7 |
| *SLITRK6* | -3.8 | -8.3 | 100% | 100% | 80% | 100% | Resistance | SLIT and NTRK-like protein 6 |
| *FOLR1* | -6.2 | -5.7 | 100% | 100% | 90% | 100% | Resistance | Folate receptor alpha |
| *VGLL1* | -6.7 | -6.2 | 90% | 100% | 90% | 100% | Resistance | Transcription cofactor vestigial-like protein 1 |
| *SFTA2* | -6.9 | -5.2 | 90% | 100% | 100% | 100% | Resistance | Surfactant-associated protein 2 |
| *MUC15* | -7.3 | -7.1 | 80% | 100% | 80% | 100% | Resistance | Mucin-15 |
| *IGFL* | -9.5 | -5.6 | 90% | 100% | 70% | 100% | Resistance | Insulin growth factor-like family member 1 |
|  |  |  |  |  |  |  |  |  |
| **Bevacizumab / Cetuximab** | | | | | | | | |
| Gene | log2.Pat | log2.PDX | Sens.Pat | Spec.Pat | Sens.PDX | Spec.PDX | Prediction | Description |
| *CLDN10* | 4.6 | 6.9 | 100% | 91% | 100% | 73% | Response | Claudin-10 |
| *CGLN* | 3.8 | 5.1 | 100% | 91% | 100% | 100% | Response | Calmegin |
| *PTK7* | -1.9 | -5.8 | 100% | 100% | 91% | 100% | Resistance | Inactive tyrosine-protein kinase 7 |
| *KLK7* | -4.9 | -5.2 | 82% | 100% | 82% | 100% | Resistance | Kallikrein-7 |
| *OTX1* | -5.3 | -3.4 | 91% | 100% | 91% | 100% | Resistance | Homeobox protein OTX1 |
| *KRT17* | -7.2 | -3.1 | 91% | 100% | 82% | 100% | Resistance | Keratin, type I cytoskeletal 17 |
| *CYP2W1* | -9.0 | -6.1 | 82% | 100% | 82% | 100% | Resistance | Cytochrome P450 2W1 |
| *KRT6A* | -10.8 | -8.1 | 91% | 100% | 82% | 100% | Resistance | Keratin, type II cytoskeletal 6A |
|  |  |  |  |  |  |  |  |  |
| **Oxaliplatin** | | | | | | | | |
| Gene | log2.Pat | log2.PDX | Sens.Pat | Spec.Pat | Sens.PDX | Spec.PDX | Prediction | Description |
| *DMBT1* | 7.0 | 5.7 | 100% | 91% | 100% | 91% | Response | Deleted in malignant brain tumors 1 protein |
| *HSD3B1* | 4.5 | 6.5 | 100% | 100% | 100% | 100% | Response | 3 beta-hydroxysteroid dehyfrogenase/Delta 5-->4isomerase type 1 |
| *FRMPD1* | 3.8 | 5.0 | 100% | 100% | 100% | 82% | Response | FERM and PDZ domain-containing protein 1 |
| *HACD1* | -2.7 | -5.2 | 100% | 100% | 100% | 100% | Resistance | Very-long-chain (3R)-3-hydroxyacyl-CoA dehydrogenase 1 |
| *SOSTDC1* | -8.8 | -7.6 | 91% | 100% | 91% | 100% | Resistance | Sclerosin domain-containing protein 1 |
|  |  |  |  |  |  |  |  |  |
| **Selumetinib** | | | | | | | | |
| Gene | log2.Pat | log2.PDX | Sens.Pat | Spec.Pat | Sens.PDX | Spec.PDX | Prediction | Description |
| *GPC2* | -1.6 | -2.6 | 100% | 100% | 90% | 100% | Resistance | Glypican-2 |
| *PALM3* | -3.5 | -3.4 | 80% | 100% | 70% | 100% | Resistance | Paralemmin-3 |
| *FAM3B* | -5.2 | -6.3 | 70% | 100% | 80% | 100% | Resistance | Protein FAM3B |
| *C6orf15* | -6.0 | -5.3 | 70% | 100% | 80% | 100% | Resistance |  |
| *ABHD12B* | -7.0 | -9.2 | 80% | 100% | 60% | 100% | Resistance | Protein ABHD12B |
| *CYP2W1* | -9.7 | -6.3 | 90% | 100% | 90% | 100% | Resistance | Cytochrome P450 2W1 |
|  |  |  |  |  |  |  |  |  |
| **Docetaxel** | | | | | | | | |
| Gene | log2.Pat | log2.PDX | Sens.Pat | Spec.Pat | Sens.PDX | Spec.PDX | Prediction | Description |
| *DSG3* | -4.9 | -4.2 | 90% | 100% | 70% | 100% | Resistance | Desmoglein-3 |
| *MYH4* | -5.2 | -4.4 | 70% | 100% | 80% | 100% | Resistance | Myosin-4 |
|  |  |  |  |  |  |  |  |  |
| **Everolimus** | | | | | | | | |
| Gene | log2.Pat | log2.PDX | Sens.Pat | Spec.Pat | Sens.PDX | Spec.PDX | Prediction | Description |
| *UNC93A* | -6.9 | -5.4 | 91% | 100% | 91% | 100% | Resistance | Protein unc-93 homolog A |
| *IGF2BP1* | -8.9 | -10.1 | 73% | 100% | 73% | 100% | Resistance | Insulin-like growth factor 2 mRNA-binding protein 1 |

**log2.Pat** – log2-fold change in patient tissue, **log2.PDX** – log2-fold change in PDX tumor, **Sens.Pat** – sensitivity in patient tissue, **Spec.Pat** – specificity in patient tissue, **Sens.PDX** – sensitivity in PDX tumor, **Spec.Pat** – specificity in PDX tumor

**Table S11: Matched predictive sequence variants for treatment response in pmCRC**

| **5-FU** | | **Resp:Res** | **PDX** | **Pat** | **PD3D** |  |  | **Trametinib** | | **Resp:Res** | **PDX** | **Pat** | **PD3D** |  |
| --- | --- | --- | --- | --- | --- | --- | --- | --- | --- | --- | --- | --- | --- | --- |
|  |  |  | 5:9 | 4:9 | 4:4 |  |  |  |  |  | 5:9 | 4:9 | 2:6 |  |
| **SYMBOL** | **Protein Position** | **Amino Acids** | **p-val PDX** | **p-val Pat** | **p-val PD3D** | **Existing Variation** |  | **SYMBOL** | **Protein Position** | **Amino Acids** | **p-val PDX** | **p-val Pat** | **p-val PD3D** | **Existing Variation** |
| PGM3 | 344 | D/N | 0.0005 | 0.0014 | 0.0357 | rs473267 |  | UNC50 | 1 | M/K | 0.002997 | 0.004662 | 0.03571 | rs1062847 |
| USP31 | 205 | I/T | 0.0005 | 0.0014 | 0.0357 | rs13339649 |  | SEMA4D | 592 | G/D | 0.002997 | 0.004662 | 0.03571 | rs45464494 |
| TBRG4 | 57 | P/L | 0.0030 | 0.0070 | 0.0357 | rs2304693 |  | ZNF638 | 1215 | V/M | 0.004995 | 0.006993 | 0.02777 | rs1804020 |
| TBRG4 | 22 | A/S | 0.0030 | 0.0070 | 0.0357 | rs2304694 |  | GPN2 | 264 | R/G | 0.02298 | 0.03186 | 0.03571 | rs3170660 |
| CLRN3 | 75 | F/I | 0.0050 | 0.0140 | 0.0357 | rs35070529 |  | POLL | 306 | L/P | 0.02298 | 0.03186 | 0.03571 | rs3730476 |
| DHX37 | 96 | M/I | 0.0050 | 0.0014 | 0.0357 | rs11558556 |  | NOB1 | 231 | R/Q | 0.02298 | 0.03497 | 0.03571 | rs3811348 |
| GSDMA | 128 | V/L | 0.0050 | 0.0014 | 0.0357 | rs7212938 |  | ARFGAP2 | 274 | S/N | 0.02747 | 0.03497 | 0.02777 | rs3740691 |
| GSDMB | 282 | G/R | 0.0050 | 0.0140 | 0.0357 | rs2305479 |  | INPP4A | 249 | T/A | 0.03097 | 0.004662 | 0.03571 | rs2278206 |
| GSDMB | 289 | P/S | 0.0050 | 0.0140 | 0.0357 | rs2305480 |  |  |  |  |  |  |  |  |
| METTL2B | 169 | E/K | 0.0050 | 0.0140 | 0.0357 | rs1065267 |  | **Oxaliplatin** | | **Resp:Res** | **PDX** | **Pat** | **PD3D** |  |
| TMPRSS2 | 160 | V/M | 0.0050 | 0.0014 | 0.0357 | rs12329760 |  |  |  |  | 2:12 | 2:11 | 0:8 |  |
| NAA40 | 42 | V/I | 0.0210 | 0.0210 | 0.0357 | rs3740637 |  | **SYMBOL** | **Protein Position** | **Amino Acids** | **p-val PDX** | **p-val Pat** | **p-val PD3D** | **Existing Variation** |
| PODXL | 194 | S/L | 0.0210 | 0.0210 | 0.0357 | rs12670788 |  | AGFG2 | 115 | M/T | 0.03297 | 0.01282 |  | rs17855473 |
| PODXL | 112 | G/S | 0.0210 | 0.0210 | 0.0357 | rs3735035 |  | C2CD2L | 264 | R/W | 0.03297 | 0.01282 |  | rs2239896 |
| TLDC1 | 145 | D/E | 0.0210 | 0.0210 | 0.0357 | rs436278 |  | FREM1 | 1203 | V/M | 0.03297 | 0.01282 |  | rs10961700 |
| TBC1D16 | 73 | T/I | 0.0230 | 0.0210 | 0.0357 | rs4889804 |  | LAMC2 | 733 | S/T | 0.03297 | 0.01282 |  | rs2296303 |
| CCDC50 | 258 | I/N | 0.0275 | 0.0140 | 0.0357 | rs2028574 |  | MRPS2 | 294 | H/R | 0.03297 | 0.01282 |  | rs3748199 |
| CCDC50 | 303 | K/R | 0.0275 | 0.0140 | 0.0357 | rs4677728 |  | SLC25A27 | 197 | I/T | 0.03297 | 0.01282 |  | rs35884480 |
| GSDMA | 130 | E/K | 0.0275 | 0.0140 | 0.0357 | rs7212944 |  | TBC1D8 | 926 | G/R | 0.03297 | 0.01282 |  | rs1062062 |
| NRBP2 | 468 | R/C | 0.0275 | 0.0140 | 0.0357 | rs72693365 |  | TNS3 | 1050 | T/A | 0.03297 | 0.01282 |  | rs61731308 |
| SART3 | 23 | D/E | 0.0275 | 0.0014 | 0.0357 | rs2072579 |  | WWOX | 153 | S/I | 0.03297 | 0.01282 |  | rs383362 |
|  |  |  |  |  |  |  |  | ERICH1 | 219 | T/I | 0.03297 | 0.03846 |  | rs61741834 |
| **Erlotinib** | | **Resp:Res** | **PDX** | **Pat** | **PD3D** |  |  | HSD3B1 | 367 | T/N | 0.03297 | 0.03846 |  | rs45609334 rs1047303 |
|  |  |  | 4:10 | 3:10 | 2:6 |  |  | NDFIP2 | 135 | A/V | 0.03297 | 0.03846 |  | rs11549502 |
| **SYMBOL** | **Protein Position** | **Amino Acids** | **p-val PDX** | **p-val Pat** | **p-val PD3D** | **Existing Variation** |  | PLEKHM2 | 32 | I/T | 0.03297 | 0.03846 |  | rs12091750 |
| CLRN3 | 75 | F/I | 0.001 | 0.0035 | 0.0357 | rs35070529 |  | TAF11 | 2 | E/K | 0.03297 | 0.03846 |  | rs13198340 |
| GSDMB | 282 | G/R | 0.001 | 0.0035 | 0.0357 | rs2305479 |  | TAF11 | 24 | T/R | 0.03297 | 0.03846 |  | rs15922 |
| GSDMB | 289 | P/S | 0.001 | 0.0035 | 0.0357 | rs2305480 |  | VSIG10 | 239-241 | EEE/E | 0.03297 | 0.03846 |  | rs72125532 |
| ASCC3 | 146 | L/F | 0.001 | 0.03497 | 0.0357 | rs9390698 |  | VSIG10 | 202 | H/Y | 0.03297 | 0.03846 |  | rs7307331 |
| FBXO3 | 221 | V/I | 0.005 | 0.01399 | 0.0357 | rs1402954 |  | ZC3HC1 | 103 | T/A | 0.03297 | 0.03846 |  | rs1464890 |
| HELZ2 | 1447 | P/L | 0.005 | 0.01399 | 0.0357 | rs3810485 |  |  |  |  |  |  |  |  |
| MUC20 | 671 | S/C | 0.005 | 0.01399 | 0.0357 | rs3762739 |  | **Everolimus** | | **Resp:Res** | **PDX** | **Pat** | **PD3D** |  |
| PGM3 | 344 | D/N | 0.005 | 0.01399 | 0.0357 | rs473267 |  |  |  |  | 3:11 | 2:11 | 0:8 |  |
| STK40 | 395 | A/T | 0.005 | 0.01399 | 0.0357 | rs3795498 |  | **SYMBOL** | **Protein Position** | **Amino Acids** | **p-val PDX** | **p-val Pat** | **p-val PD3D** | **Existing Variation** |
| USP31 | 205 | I/T | 0.005 | 0.01399 | 0.0357 | rs13339649 |  | EML3 | 14 | A/V | 0.01099 | 0.03846 |  | rs12808829 |
| WASHC2C | 162 | T/M | 0.005 | 0.01399 | 0.0357 | rs199748321 |  | SUMF1 | 398 | F/L | 0.03297 | 0.03846 |  | rs795734 |
| LRR1 | 96 | I/N | 0.005 | 0.03497 | 0.0357 | rs17121605 |  | ZNF333 | 11 | E/A | 0.03297 | 0.03846 |  | rs75269766 |
| LRR1 | 229 | R/W | 0.005 | 0.03497 | 0.0357 | rs7148147 |  |  |  |  |  |  |  |  |
| CCDC50 | 258 | I/N | 0.01099 | 0.0035 | 0.0357 | rs2028574 |  | **Docetaxel** | | **Resp:Res** | **PDX** | **Pat** | **PD3D** |  |
| CCDC50 | 303 | K/R | 0.01099 | 0.0035 | 0.0357 | rs4677728 |  |  |  |  | 3:11 | 3:10 | 2:6 |  |
| GSDMA | 130 | E/K | 0.01099 | 0.0035 | 0.0357 | rs7212944 |  | **SYMBOL** | **Protein Position** | **Amino Acids** | **p-val PDX** | **p-val Pat** | **p-val PD3D** | **Existing Variation** |
| NRBP2 | 468 | R/C | 0.01099 | 0.0035 | 0.0357 | rs72693365 |  | ALDH3A1 | 134 | S/A | 0.01099 | 0.01399 | 0.03571 | rs887241 |
| SART3 | 23 | D/E | 0.01099 | 0.01399 | 0.0357 | rs2072579 |  | GPN2 | 264 | R/G | 0.02747 | 0.03497 | 0.03571 | rs3170660 |
| AFMID | 36 | Q/R | 0.01099 | 0.03846 | 0.0357 | rs72897838 |  | KIF16B | 1207 | G/C | 0.02747 | 0.03497 | 0.03571 | rs2328020 |
| CCDC50 | 156 | M/T | 0.01099 | 0.03846 | 0.0357 | rs293813 |  |  |  |  |  |  |  |  |
| DHX57 | 308 | S/F | 0.01099 | 0.03846 | 0.0357 | rs11893062 |  | **Selumetinib** | | **Resp:Res** | **PDX** | **Pat** | **PD3D** |  |
| FARP1 | 593 | H/Y | 0.01099 | 0.03846 | 0.0357 | rs61730892 |  |  |  |  | 4:10 | 3:10 | n.t. |  |
| IQCK | 132 | L/P | 0.01099 | 0.03846 | 0.0357 | rs7191155 |  | **SYMBOL** | **Protein Position** | **Amino Acids** | **p-val PDX** | **p-val Pat** | **p-val PD3D** | **Existing Variation** |
| KLC2 | 320 | P/S | 0.01099 | 0.03846 | 0.0357 | rs2276036 |  | DEF8 | 197 | M/I | 0.004995 | 0.01399 |  | rs8166 |
| KRAS | 12 | G/C | 0.01099 | 0.03846 | 0.0357 | rs121913530 |  | BRD8 | 384 | T/M | 0.004995 | 0.03497 |  | rs11750814 |
| MIIP | 142 | P/S | 0.01099 | 0.03846 | 0.0357 | rs11588712 |  | FAM160A2 | 619 | R/L | 0.004995 | 0.03846 |  | rs3750943 |
| QRICH1 | 771 | N/S | 0.01099 | 0.03846 | 0.0357 | rs61729488 |  | NOL10 | 485 | D/N | 0.01099 | 0.03497 |  | rs2287059 |
| RLF | 1296 | Q/E | 0.01099 | 0.03846 | 0.0357 | rs34141181 |  | BAZ2B | 1145 | S/N | 0.01099 | 0.03846 |  | rs415793 |
| RNF123 | 854 | R/H | 0.01099 | 0.03846 | 0.0357 | rs34823813 |  | BBS12 | 467 | D/N | 0.01099 | 0.03846 |  | rs13135778 |
| RNF43 | 104 | P/L | 0.01099 | 0.03846 | 0.0357 | rs2680701 |  | CCPG1 | 196 | A/G | 0.01099 | 0.03846 |  | rs117236526 |
| SH3BP1 | 587 | A/V | 0.01099 | 0.03846 | 0.0357 | rs12483880 |  | CKAP2 | 435 | E/K | 0.01099 | 0.03846 |  | rs41292820 |
| SLC38A10 | 477 | K/R | 0.01099 | 0.03846 | 0.0357 | rs35546507 |  | CLCN7 | 360 | V/M | 0.01099 | 0.03846 |  | rs12926089 |
| SLC38A10 | 594 | E/D | 0.01099 | 0.03846 | 0.0357 | rs55872261 |  | CROCC | 1038 | V/M | 0.01099 | 0.03846 |  | rs41272737 |
| TP53I3 | 230 | T/A | 0.01099 | 0.03846 | 0.0357 | rs10209238 |  | CROCC | 1672 | S/G | 0.01099 | 0.03846 |  | rs56278097 |
| TRAF3IP1 | 276 | M/L | 0.01099 | 0.03846 | 0.0357 | rs3739070 |  | CROCC | 1399 | G/R | 0.01099 | 0.03846 |  | rs78888579 |
| UBE3D | 192 | V/M | 0.01099 | 0.03846 | 0.0357 | rs7739323 |  | IL17RC | 297 | P/L | 0.01099 | 0.03846 |  | rs115419420 |
| USP19 | 36 | D/H | 0.01099 | 0.03846 | 0.0357 | rs11552724 |  | LAMA5 | 1626 | H/Y | 0.01099 | 0.03846 |  | rs875379 |
| ZCCHC14 | 112 | Y/H | 0.01099 | 0.03846 | 0.0357 | rs78839939 |  | LIG4 | 3 | A/V | 0.01099 | 0.03846 |  | rs1805389 |
| ZNF417 | 168 | P/S | 0.01099 | 0.03846 | 0.0357 | rs142466487 |  | MATN2 | 717 | T/M | 0.01099 | 0.03846 |  | rs2255317 |
| TBRG4 | 57 | P/L | 0.01499 | 0.03497 | 0.0357 | rs2304693 |  | MROH1 | 483 | I/V | 0.01099 | 0.03846 |  | rs112996909 |
| TBRG4 | 22 | A/S | 0.01499 | 0.03497 | 0.0357 | rs2304694 |  | NCEH1 | 71 | K/Q | 0.01099 | 0.03846 |  | rs2302815 |
| DHX37 | 96 | M/I | 0.04096 | 0.01399 | 0.0357 | rs11558556 |  | PATJ | 303 | G/R | 0.01099 | 0.03846 |  | rs3762321 |
| GSDMA | 128 | V/L | 0.04096 | 0.01399 | 0.0357 | rs7212938 |  | PCSK9 | 14-15 | -/L | 0.01099 | 0.03846 |  | rs371488778 |
| TMPRSS2 | 160 | V/M | 0.04096 | 0.01399 | 0.0357 | rs12329760 |  | PCSK9 | 53 | A/V | 0.01099 | 0.03846 |  | rs11583680 |
| TTC3 | 118 | M/T | 0.04096 | 0.01399 | 0.0357 | rs1053808 |  | PTPN12 | 576 | E/K | 0.01099 | 0.03846 |  | rs2230602 |
| TTC3 | 1029 | D/H | 0.04096 | 0.01399 | 0.0357 | rs1053966 |  | RFX5 | 238 | P/R | 0.01099 | 0.03846 |  | rs2233854 |
| BCAS1 | 491 | S/P | 0.04096 | 0.03846 | 0.0357 | rs1055246 |  | SEL1L | 691 | V/I | 0.01099 | 0.03846 |  | rs1051193 |
| PPP1R15A | 597 | T/A | 0.04096 | 0.03846 | 0.0357 | rs500079 |  | SUCLG2 | 425 | V/I | 0.01099 | 0.03846 |  | rs902320 |
| STK31 | 232 | G/E | 0.04096 | 0.03846 | 0.0357 | rs4722266 |  | THAP12 | 355 | I/V | 0.01099 | 0.03846 |  | - |
| WWC1 | 772-773 | VE/V | 0.04096 | 0.03846 | 0.0357 | rs111457550 |  | TMF1 | 798 | D/H | 0.01099 | 0.03846 |  | rs1532918 |
|  |  |  |  |  |  |  |  | TUBGCP3 | 428 | R/H | 0.01099 | 0.03846 |  | rs41288616 |
| **Cetuximab** | | **Resp:Res** | **PDX** | **Pat** | **PD3D** |  |  | CCSER2 | 84 | N/S | 0.01499 | 0.03497 |  | rs3814205 |
|  |  |  | 3:10 | 2:10 | 0:8 |  |  | COG2 | 738 | * | 0.01499 | 0.03497 |  | rs1051038 |
| **SYMBOL** | **Protein Position** | **Amino Acids** | **p-val PDX** | **p-val Pat** | **p-val PD3D** | **Existing Variation** |  | NAAA | 356 | I/N | 0.01499 | 0.03497 |  | rs7686066 |
| SUCLG2 | 425 | V/I | 0.00275 | 0.01282 |  | rs902320 |  | SPOUT1 | 130 | T/R | 0.01499 | 0.03497 |  | rs6478854 |
| FANCA | 1045 | S/F | 0.01099 | 0.01282 |  | rs17233497 |  | STOX1 | 498 | E/D | 0.01499 | 0.03497 |  | rs10509305 |
| ABCB1 | 21 | N/D | 0.01099 | 0.03846 |  | rs9282564 |  | TBC1D13 | 114 | V/A | 0.01499 | 0.03497 |  | rs1572912 |
| FANCA | 1285 | T/A | 0.01099 | 0.03846 |  | rs9282681 |  | TOPBP1 | 1037 | N/S | 0.01499 | 0.03497 |  | rs10935070 |
| IRAK4 | 225 | A/T | 0.01099 | 0.03846 |  | rs4251545 |  | ZFP91 | 207 | S/G | 0.01499 | 0.03497 |  | rs8373 |
| MAVS | 93 | Q/E | 0.01099 | 0.03846 |  | rs17857295 |  | ADGRG1 | 106 | S/R | 0.04096 | 0.01399 |  | rs1801257 |
| MC1R | 92 | V/M | 0.01099 | 0.03846 |  | rs2228479 |  | ASCC3 | 146 | L/F | 0.04096 | 0.03497 |  | rs9390698 |
| NDOR1 | 257 | V/I | 0.01099 | 0.03846 |  | rs62587579 |  | ARHGEF10L | 1175 | I/V | 0.04096 | 0.03846 |  | rs2270976 |
| PUS7L | 264 | K/E | 0.01099 | 0.03846 |  | rs1057190 |  | EFHB | 57-58 | -/P | 0.04096 | 0.03846 |  | rs878999429 |
| SENP7 | 46 | K/Q | 0.01099 | 0.03846 |  | rs6809436 |  | ERCC6L2 | 7 | P/S | 0.04096 | 0.03846 |  | rs56108623 |
| SKAP2 | 202 | A/S | 0.01099 | 0.03846 |  | rs1129771 |  | FANCA | 1045 | S/F | 0.04096 | 0.03846 |  | rs17233497 |
| SLC28A2 | 22 | P/L | 0.01099 | 0.03846 |  | rs11854484 |  | UTP6 | 69 | Q/R | 0.04096 | 0.03846 |  | rs3760454 |
| TCF25 | 391 | R/Q | 0.01099 | 0.03846 |  | rs57940434 |  |  |  |  |  |  |  |  |
| TMEM132A | 824 | E/K | 0.01099 | 0.03846 |  | rs55920775 |  |  |  |  |  |  |  |  |
| TOMM34 | 185 | H/R | 0.01099 | 0.03846 |  | rs1804644 |  |  |  |  |  |  |  |  |
| SMPD2 | 204 | S/X | 0.03297 | 0.01282 |  | rs35444917 |  |  |  |  |  |  |  |  |
| AKIP1 | 143 | T/M | 0.03297 | 0.03846 |  | rs2016844 |  |  |  |  |  |  |  |  |
| FBXO7 | 6 | G/E | 0.03297 | 0.03846 |  | rs9621461 |  |  |  |  |  |  |  |  |
| MICAL1 | 12 | A/T | 0.03297 | 0.03846 |  | rs4946977 |  |  |  |  |  |  |  |  |
| MTERF4 | 151 | L/V | 0.03297 | 0.03846 |  | rs2240539 |  |  |  |  |  |  |  |  |
| MTERF4 | 45 | T/A | 0.03297 | 0.03846 |  | rs3796093 |  |  |  |  |  |  |  |  |
| RILPL1 | 2 | T/A | 0.03297 | 0.03846 |  | - |  |  |  |  |  |  |  |  |
| RMND5B | 5 | S/G | 0.03297 | 0.03846 |  | - |  |  |  |  |  |  |  |  |
| TRIM47 | 187 | R/W | 0.03297 | 0.03846 |  | rs4600514 |  |  |  |  |  |  |  |  |
| ZACN | 281 | Q/* | 0.03297 | 0.03846 |  | rs1043149 |  |  |  |  |  |  |  |  |
| ZNF285 | 381 | G/R | 0.03297 | 0.03846 |  | rs12610859 |  |  |  |  |  |  |  |  |
| ZNF358 | 119 | K/Q | 0.03297 | 0.03846 |  | rs78214445 |  |  |  |  |  |  |  |  |
| ZNF721 | 730 | R/L | 0.03297 | 0.03846 |  | rs61793704 |  |  |  |  |  |  |  |  |
| ZWILCH | 230 | S/G | 0.03297 | 0.03846 |  | rs11071896 |  |  |  |  |  |  |  |  |

**Resp:Res** – number of responding vs. resistant models used for prediction analysis;

**Table S12: Validation of improved response of pmCRC models to combination therapy with PARP inhibitors**

| **Biomarker (Pat, TPM)** | **5-FU** | **CRC-05A** | **CRC-09** | **CRC-19A** | **CRC-28B** | **CRC-55A** | **CRC-55B** |
| --- | --- | --- | --- | --- | --- | --- | --- |
| PCDHGB2 | ROC cut-off = 3.40 | 0.52 | 5.14 | 1.21 | 0.95 | 0.76 | 0.61 |
| EPOP | ROC cut-off = 2.69 | 3.52 | 1.31 | 3.23 | 3.38 | 6.5 | 5.62 |
| ATP6V1C2 | ROC cut-off = 1.44 | 3.12 | 0.92 | 2.25 | 2.03 | 4.48 | 3.12 |
| SLC7A5 | ROC cut-off = 21.96 | 50.00 | 2.6 | 26.69 | 130.02 | 187.59 | 161.38 |
| SFTA2 | ROC cut-off = 7.40 | 80.98 | 0.34 | 11.84 | 538.57 | 241.38 | 233.42 |
| **Monotherapy response** | PDX *in vivo* | low | high | low | low | low | low |
|  | PDX cells *in vitro* | low | high | low | low | (high) | (high) |
| **Improvement in**  **combination with Olaparib** | | **yes** | no | **yes** | **yes** | (no) | (no) |
| **Monotherapy response** | PD3D (1st exp) |  |  | high | high | low | low |
|  | PD3D (2nd exp) |  |  | n.t. | high | low | n.t. |
| **Improvement in**  **combination with Olaparib** | |  |  |  | no | **yes** |  |
|  |  |  |  |  |  |  |  |
| **Biomarker (Pat, TPM)** | **Trametinib** | **CRC-05A** | **CRC-09** | **CRC-21** | **CRC-28B** | **CRC-55A** | **CRC-55B** |
| CYP4X1 | ROC cut-off = 2.43 | 21.34 | 5.19 | 1.93 | 1.17 | 1.79 | 1.53 |
| ERP27 | ROC cut-off = 2.54 | 0.86 | 1.3 | 2.06 | 6.44 | 4.14 | 6.73 |
| H2AFY2 | ROC cut-off = 12.80 | 2.08 | 12.46 | 13.13 | 25.53 | 38.94 | 27.24 |
| ADGRF4 | ROC cut-off = 2.69 | 2.41 | 0.22 | 2.97 | 10.84 | 14.13 | 10.09 |
| BAMBI | ROC cut-off = 19.78 | 18.59 | 7.22 | 14.52 | 22.46 | 91.61 | 66.88 |
| FOXH1 | ROC cut-off = 0.23 | 0.11 | 0.03 | 0.91 | 3.33 | 2.23 | 1.49 |
| IL36RN | ROC cut-off = 0.37 | 0.26 | 0.04 | 0.47 | 0.80 | 9.01 | 8.33 |
| SLC30A10 | ROC cut-off = 0.04 | 0.03 | 0.03 | 0.18 | 1.03 | 0.13 | 0.08 |
| TMPRSS11E | ROC cut-off = 0.20 | 0.09 | 0.05 | 1.14 | 2.98 | 0.56 | 0.77 |
| **Monotherapy response** | PDX *in vivo* | high | high | low | low | low | low |
|  | PDX cells *in vitro* | high | high | low | n.t. | low | low |
| **Improvement in**  **combination with Olaparib** | | no | no | **yes** |  | **yes** | **yes** |
| **Monotherapy response** | PD3D (1st exp) |  |  | low | low | low | low |
|  | PD3D (2nd exp) |  |  | low | low | low | n.t. |
| **Improvement in**  **combination with Olaparib** | |  |  | **yes** | **yes** | **yes** |  |

**Pat** – pmCRC patient metastasis; **TPM** – transcripts per million; **ROC** – receiver operator curve based; **exp** – experiment;

**n.t.** – not tested
